# Transgenic Expression of *Haemonchus contortus* Cytochrome P450 *Hco-cyp-13A11* Decreases Susceptibility to Particular but Not All Macrocyclic Lactones in the Model Organism *Caenorhabditis elegans*

**DOI:** 10.3390/ijms23169155

**Published:** 2022-08-15

**Authors:** Natalie Jakobs, Esra Yilmaz, Jürgen Krücken

**Affiliations:** Institute for Parasitology and Tropical Veterinary Medicine, Freie Universität Berlin, 14163 Berlin, Germany

**Keywords:** macrocyclic lactones, drug resistance, cytochrome P450, drug metabolism, parasitology, parasite metabolism

## Abstract

The number of reported macrocyclic lactones (ML) resistance cases across all livestock hosts is steadily increasing. Different studies in the parasitic nematode *Haemonchus contortus* assume the participation of cytochrome P450s (Cyps) enzymes in ML resistance. Still, functional data about their individual contribution to resistance or substrate specificity is missing. Via microinjection, transgenic *Caenorhabditis elegans* expressing HCON_00141052 (*transgene-Hco-cyp-13A11*) from extrachromosomal arrays were generated. After 24 h of exposure to different concentrations of ivermectin (IVM), ivermectin aglycone (IVMa), selamectin (SEL), doramectin (DRM), eprinomectin (EPR), and moxidectin (MOX), motility assays were performed to determine the impact of the *H. contortus* Cyp to the susceptibility of the worms against each ML. While *transgene*-*Hco-cyp-13A11* significantly decreased susceptibility to IVM (four-fold), IVMa (2-fold), and SEL (3-fold), a slight effect for DRM and no effect for MOX, and EPR was observed. This substrate specificity of *Hco-cyp-13A11* could not be explained by molecular modeling and docking studies. Hco-Cyp-13A11 molecular models were obtained for alleles from isolates with different resistance statuses. Although 14 amino acid polymorphisms were detected, none was resistance specific. In conclusion, Hco-cyp-13A11 decreased IVM, IVMa, and SEL susceptibility to a different extent, but its potential impact on ML resistance is not driven by polymorphisms.

## 1. Introduction

Anthelmintic therapy remains the most important management strategy to treat parasitic nematode infections in humans and livestock. Due to their broad-spectrum activity and high efficacy, macrocyclic lactones (MLs), including ivermectin (IVM), moxidectin (MOX), selamectin (SEL), eprinomectin (EPR), and doramectin (DRM), represent the most widely used drug class [1]. Both subfamilies of MLs, the milbemycins (MOX and milbemycin oxime) and the avermectins (IVM, SEL, EPR, and DRM), share the core structure, a 16-member macrocyclic lactone ring fused with a benzofuran and a spiroketal moiety. Structural differences between the subfamilies relate to the presence and absence of specific substituents [1].

The extensive and inappropriate use of these drugs has led to the worldwide emergence of ML resistance [2,3]. This increasingly impairs the treatment of trichostrongyloid parasitic nematodes, particularly *Haemonchus contortus*, one of the most pathogenic gastrointestinal nematodes infecting small ruminants [4]. Intensive research on ML resistance revealed a multi-genic mechanism, including target-site [5,6,7,8] and non-target-site associated changes [9,10,11,12]. The latter mainly include pharmacokinetic-related defense via drug biotransformation enzymes [12,13] and efflux transporters [11,14,15,16,17].

Drug metabolism can generally be divided into two phases. In phase I, xenobiotics can undergo modification via oxidation, reduction, or hydrolysis reactions to insert or uncover hydrophilic groups. Depending on the hydrophilicity of the resulting products, phase II can follow, which involves conjugation with endogenous compounds such as glucuronic acid, glucose, or glutathione [18]. Although expression changes of genes encoding phase II UDP-glycosyltransferases (UGTs) between susceptible and resistant *H. contortus* isolates were reported for the benzimidazole albendazole [19], the contribution of UGTs to ML resistance has to date not been registered. In contrast, IVM-selected *Caenorhabditis elegans* strains induced the overexpression of two-phase II glutathione *S*-transferases [20].

The largest family of biotransformation enzymes comprises cytochrome P450 enzymes (Cyps). These phase I enzymes are present in almost all living organisms and contribute to drug resistance via various catalytic reactions [21]. The human genome encodes 57 Cyps, with five Cyps dominating drug metabolism [22], and within this group, Cyp-3A4 is present in the largest quantities within the human body [23]. The *C. elegans* genome even encodes 80 Cyps [24]. In particular, the members of the family Cyp35 were shown to be xenobiotically inducible [25,26]. Within the *H. contortus* genome, a smaller number of 42 Cyps was identified [27] without the extensive gene duplications observed in *C. elegans* [24]. A study by Yilmaz et al. further suggested differences in constitutive expression of Cyps for *H. contortus* isolates with different resistance status since the Cyp34/35 ortholog (HCON_00022640) was identified to be constitutively higher expressed in the multi-resistant *H. contortus* White River (WR) isolate [28].

Investigating IVM metabolite formation in mammalian liver microsomes has already elucidated the contribution of human Cyp isoforms to IVM metabolism, particularly cytochrome P450 3A4 [29,30,31]. Inhibition of human liver-derived Cyp-3A4 with troleandomycin specifically diminished IVM biotransformation by >90, thereby revealing Cyp-3A4 as a critical enzyme in IVM biotransformation [30]. The importance of Cyp-3A4 in ML metabolism was also confirmed by conducting in vitro studies analyzing MOX metabolism by various mammalian species. Cyp-3A4 hydroxylates IVM and MOX at the alkyl side chains of the spiroketal moiety [30,32]. Although in vitro metabolism of MOX has also been reported in adult *H. contortus*, neither in vitro nor ex vivo IVM biotransformation products were found [33,34]. Differences in oxidative metabolism between ML-susceptible and -resistant *H. contortus* by measuring Cyp-mediated transformation of model substrates were not identified [35].

Nevertheless, several studies supported the potential contribution of Cyps to ML resistance, particularly for IVM. When inhibiting Cyp activity with the widely used inhibitor piperonyl butoxide and simultaneous exposure to IVM, ML-susceptible and -resistant *Cooperia oncophora* and *Osteragia ostertagi* showed complete inhibition of larval development and a significant reduction in larval migration [36]. Furthermore, IVM- and MOX-selected *C. elegans* strains showing decreased ML sensitivity displayed the constitutive overexpression of various Cyps [20]. While overexpression of *Cel-cyp-14A2* and *Cel-cyp-14A5* was observed for IVM and MOX selected strains, *Cel-cyp-35A1* appeared to be specific for MOX and *Cel-cyp-37B1* specific for IVM selected strains [20]. A later study by Yilmaz et al. (2019) excluded *Cel-cyp-14A5* in ML metabolism using a loss-of-function variant of *C. elegans cyp-14A5*. In addition, in adults of the drug-susceptible *H. contortus* isolate ISE only minorly, and no significant IVM-inducible changes in the expression of different candidate Cyps were observed [19].

By investigating the genetic bases of ML resistance, whole-genome analysis of a triple resistant *Teladorsagia circumcincta* isolate introgressed into a susceptible background elucidated various resistance-associated genes. Besides differential expression of efflux transporters, the TELCIR_18530 ortholog of the *C. elegans* Cyp-13A subfamily was approximately 20-fold overexpressed in resistant worms (see S7 Table in [16]). The *C. elegans* Cyp13A subfamily has already been demonstrated to be inducible by the xenobiotic rifampicin [37]. However, analysis of constitutive Cyp expression in different life-cycle stages of the drug-susceptible *H. contortus* isolate ISE revealed the highest HCON_00141052 (orthologue of *Cel-cyp-13A11 and Cel-cyp-13A12*) expression in eggs. In contrast, low or no expression of this Cyp has been observed in L4 and adult worms [24]. Whether *Hco-cyp-13A11* can potentially contribute to an increased ML resistance level in *H. contortus* remains unclear.

Therefore, the present work investigated the impact of *Hco-cyp-13A11* on ML susceptibility by transgenic expression in the model organism *C. elegans*. To examine whether different structural features of MLs have effects on the substrate specificity of *Hco-cyp-13A11*, IVM, IVMa, MOX, EPR, SEL, and DRM were tested. The exon sequence of *Hco-cyp-13A11* was compared between *H. contortus* isolates with different resistance statuses to identify single nucleotide polymorphisms correlating with ML resistance. Their potential impact regarding changes in ML binding of *Hco-cyp-13A11* was elucidated by molecular modeling and docking.

## 2. Results

### 2.1. Caenorhabditis elegans Motility Assays with Ivermectin, Moxidectin, Ivermectin Aglycone, Doramectin, Selamectin, and Eprinomectin

*Haemonchus contortus cyp-13A11* was expressed in the model organism *C. elegans* N2 Bristol wild-type (WT) by introducing extrachromosomal transgene arrays using microinjection of a plasmid construct to determine the impact of *Hco-cyp-13A11* on the susceptibility towards various MLs via drug metabolism (Figure S1). The candidate gene sequence was derived from the highly IVM and BZ resistant WR isolate. To identify transgenic worms, a plasmid driving pharyngeal *gfp* expression in *C. elegans* was co-injected. Expression of the Cyp was under the control of the *C. elegans* intestine epithelium-specific *gut esterase 1* promotor (*ges-1p*) [38], since IVM uptake was demonstrated to occur via active pharyngeal pumping through the gut epithelium [39] and xenobiotic metabolism is primarily assumed to take part in the gut epithelium of nematodes [40]. The WT was used as the control line since Gerhard et al. [39] did not observe significant differences between WT and mock-transduced nematodes using almost identical constructs.

**Figure 1:**
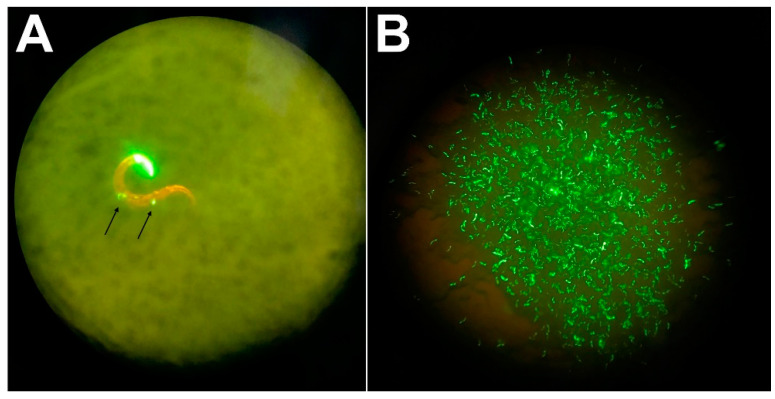
Fluorescence photographs of a single transgenic line on an NGM-agar plate. (**A**). An individual Hco-cyp-13A11 adult transgene *C. elegans* expressing GFP in the pharynx with transgenic progeny (black arrows). (**B**). Population of adult GFP positive Hco-cyp-13A11 transgene *C. elegans*.

The transgenic line showed a semi-stable transmission pattern with GFP expression in the offspring, varying between 50 and 70%. In addition, no noticeable variability of fluorescence intensity was observed between GFP-positive individuals (Figure 1). For motility assays, only individuals expressing GFP in the pharynx were used. *Escherichia coli* OP50 as a food source was added, with an approximate OD_600_ of 0.5 as recently described to stimulate drug uptake by the pharynx [39]. The expression of the candidate *Hco-cyp-13A11* was confirmed by RT-PCR, targeting the full-length sequence.

**Figure 2:**
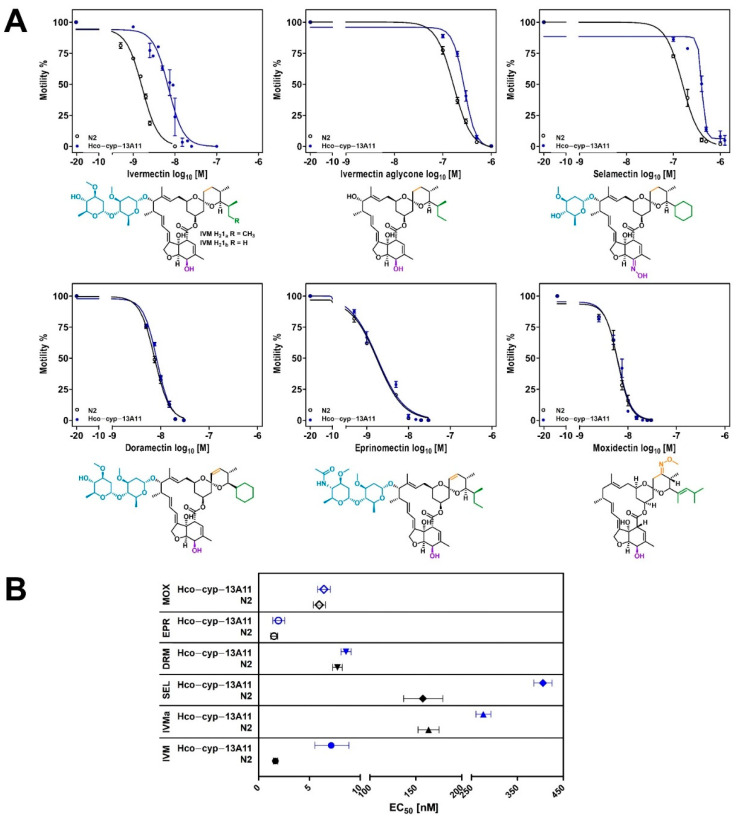
Modulation of macrocyclic lactone susceptibility in *Caenorhabditis elegans* by transgenic expression of *Hco-cyp-13A11*. (**A**) Concentration-response curves to ivermectin, ivermectin aglycone, selamectin, doramectin, eprinomectin, and moxidectin of the N2 wild-type and the *transgene-Hco-cyp-13A11* under control of the gut epithelium-specific promotor *ges-1*. The corresponding chemical drug structure is presented below the graph and highlighted according to the structural differences to the other macrocyclic lactones. **Blue**: carbohydrate moiety; **purple**: hydroxyl/oxime substituent; **green**: alkyl-side chain; **yellow**: altered spiroketal function. Adult worms were incubated for 24 h in S-medium with *Escherichia coli* OP-50 containing different drug concentrations. The motility of individual worms was assessed as body bends per minute. The negative control, only containing 1% DMSO, was set to 10^-20^ M before log_10_ transformation of concentrations. Values represent the means ± standard error of the mean of at least four biological replicates. At least 12 worms per concentration were counted for each replicate, and the mean normalized to the DMSO mean. The bottom and top values for four-parameter logistic regression were constrained to values between 0 and 100%. (**B**) Forest plot visualizing resulting EC_50_ values [nM], corresponding 95% confidence interval for the N2 control (**black**) and the transgenic *Hco-cyp-13A11 C. elegans* strain (**blue**) from concentration-response curves presented in (**A**). *Hco-cyp-13A11* genotype is *gutCyp-13A11Ex1* [*Cel-ges-1p::Hco-cyp-13A11::FLAG::Cel-unc-54_3′-UTR; Cel-myo-2p::gfp::Cel-unc-54_3′UTR*]; control strain is N2 Bristol wild-type; IVM: ivermectin; MOX: moxidectin; EPR: eprinomectin; IVMa: ivermectin aglycone; SEL: selamectin; DRM: doramectin.

A statistically significant increase (*p* < 0.0001) in EC_50_ values was observed in thrashing assays investigating IVM, IVMa, and SEL for worms transgenic for *Hco-cyp-13A11* (*transgene-Hco-cyp-13A11*) compared to the N2 wild-type control. The highest increase in EC_50_ value was observed for IVM with an approximate fold-change of 4.3, followed by 2.6 for SEL and IVMa with a 1.7-fold change (Figure 2 and Table 1). In contrast, exposure to DRM led only to a slight effect (*p* = 0.005), and MOX and EPR did not lead to significant differences in concentration-response curves between the transgenic and the WT lines.

### 2.2. Comparison of Hco-cyp-13A11 Sequences between Susceptible and Resistant H. contortus Isolates to Identify Potential Macrocyclic Lactone Resistance-Associated Single-Nucleotide Polymorphisms (SNPs)

The encoded candidate gene sequence of *Hco-cyp-13A11* was compared between two susceptible (inbred-susceptible Edinburgh (ISE) and McMaster (McM)) and two ML resistant (WR and Berlin-selected isolate (BSI)) isolates to identify potential resistance-associated single nucleotide polymorphisms (SNPs). Although a reference sequence has already been published for ISE [41], the Cyp gene for this isolate was analyzed analogously to the others using Sanger sequencing. Genomic DNA was extracted from *H. contortus* third-stage larvae to amplify sequences spanning one to multiple exons of *Hco-cyp-13A11*. Protein coding sequences (CDS) were finally assembled according to the *Hco-cyp-13A11* published ISE sequence [42] and used for analysis. Compared to the gene sequence published, the multiple sequence alignment (Figure S3) revealed complete CDSs for all isolates. The *Hco-cyp-13A11* sequences obtained for ISE, McM, and BSI corresponded to a 1555 base pair (bp) and the WR sequence to a 1549 bp open reading frame. Comparing the sequenced and reference ISE sequences, differences in 12 nucleotide positions were identified. The comparison of the two ISE sequences with the sequences obtained from the other isolates revealed two SNPs for McM, eleven for BSI, and 31 SNPs and six deletions for WR (Figure S3).

To further elucidate the impact of each SNP on potential peptide and protein structure alterations, the translated amino acid (AA) sequences were considered. The *cyp-13A11* polypeptide contains 517 residues for ISE, McM, and BSI, whereas the deletion of two adjacent codons (nucleotide positions from 1065 to 1070) encoding codons CAA and CAG in the genomic sequence reduced the number to 515 AAs for the WR isolate. The calculated molecular weights ranged between 59.4–59.6 kDa and corresponding theoretical pI values between 8.6 and 9.1 (Table 2). Post-translational modifications or functional motifs were predicted by ExPASy–ScanProsite, revealing the same cysteine heme-iron-binding motif FGlGPRQCIG (residues 454–463 for ISE, McM, BSI; residues 452–461 for McM) for all analyzed Cyps. This motif is identical to the orthologous *C. elegans cyp-13A11* and *cyp-13A12* heme-binding sites (Table 2). However, functionally or structurally validated data such as crystal structures are currently unavailable for the *C. elegans* orthologous Cyps. Keller et al. (2014) revealed that *Cel-cyp-13A12* regioselectively metabolizes arachidonic acid when expressed in insect cells, and it was proposed to act as a polyunsaturated fatty acid epoxygenase. The most closely related human orthologous gene to *Cel-cyp-13A12* (32% AA identity) and *Hco-cyp-13A11* (28% AA identity) based on a BLAST search represents Cyp-3A4.

**Figure 3:**
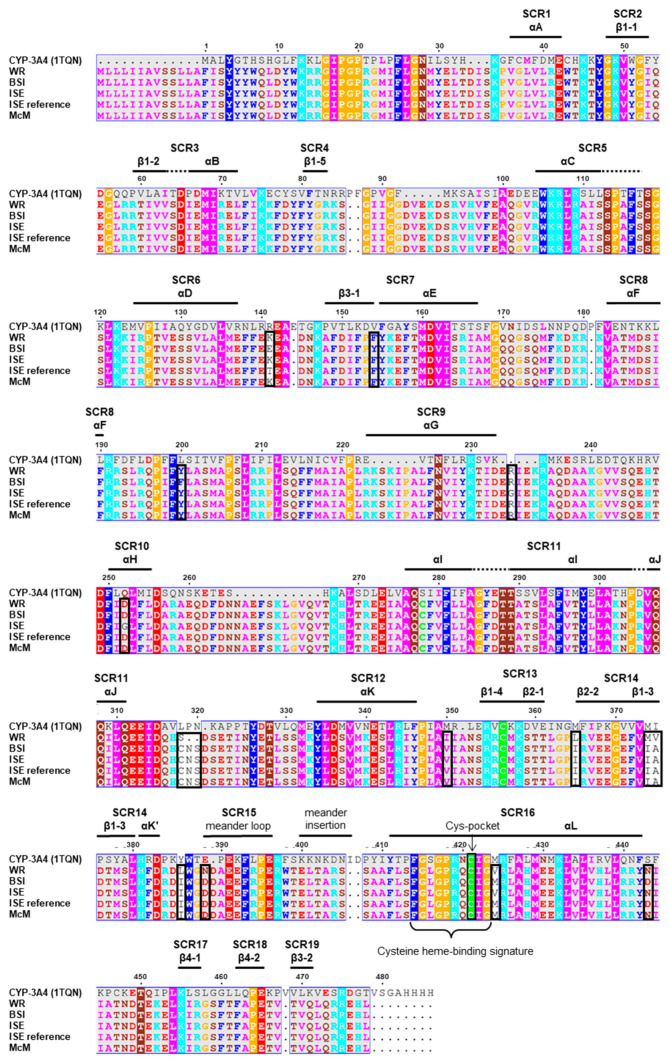
A multiple peptide sequence alignment of Hco-Cyp-13A11 from different *Haemonchus contortus* isolates, including the ISE reference sequence (HCON_00141052) [42] and human Cyp-3A4. The Hco-Cyp-13A11 amino acid sequence was predicted using ExPASy–Translate, and the alignment was obtained by Clustal Omega (version 1.2.4) [58]. Secondary structure elements were predicted with ESPript (version 3.0) [59] using the human Cyp-3A4 [43] (PDB: 1TQN), and residues were colored according to their physicochemical properties by ESPript. The annotation of structurally conserved regions (SCR) is indicated according to the multiple sequence alignment of human Cyp structures [48]. Numbers above the alignment refer to positions in the human Cyp-3A4. The cysteine heme-binding signature was predicted by ExPASy–ScanProsite. Black boxes highlight amino acid variation between different isolates for the specific position.

The human Cyp-3A4 is the most abundant Cyp in the human liver and small intestine and oxidizes structurally diverse substrates [41,43]. Its importance in drug metabolism and pharmacologic drug development fostered structure elucidation by crystallography [43,44,45,46,47]. Using the superposition of different Cyp structures from various species, Cyps have been described to contain 19 structurally conserved regions (SCRs), including defined secondary structure elements such as alpha helices αA-L and beta sheets β1-4 [48]. Assignment of a multiple sequence alignment to a crystal structure of the human Cyp-3A4 was conducted using ESPript (Figure 3) to identify *Hco-cyp-13A11* secondary structure elements. As a PDB reference, the entry 1TQN was chosen since it is the only Cyp-3A4 structure available that includes the cofactor protoporphyrin containing an iron(II) without binding other substrates or inhibitors [43]. Comparing the SCRs between the *Haemonchus* and human Cyp, the *Hco-cyp-13A11* residues for all isolates matched 38% to the Cyp-3A4 sequence. The highest residue coverages were observed for SCRs 2 (66%), 5 (57%), 11 (50%), 12 (53%), 15 (62%) and 16 (47%) (Figure 3). SCR11 αI and SCR16 αL belong to a conserved four-helix bundle which forms together with the SCR11 αJ and SCR12 αK helices and the SCR15 meander the conserved core of the Cyp [49]. SCR16 comprises the CIG motif located in the cysteine pocket serving as a ligand to the heme iron [49], which is identical between the *Haemonchus* and human Cyp. The second conserved motif is ExxR, which is present at the C-terminal end of the SCR12 αK [48,49]. While the Cyp-3A4 shows a threonine at the second position in this motif (ETLR), *Hco-cyp-13A11* encodes a serine (ESLR) (Figure 3). The rest of the SCRs showed identities < 35%, while SCR4 and 19 share no residues with Cyp-3A4. The meander insertion proposed to form a reductase interacting surface [50] showed no AA identity but an identical length of six AAs for Cyp-3A4 and *Hco-cyp-13A11* (Figure 3).

In total, 14 amino acid polymorphisms were observed between all analyzed isolates. Six positions were located in the SCRs 7, 10, 14, 15, and 16 based on the multiple peptide sequence alignment (Figure 3). The variations within the SCRs occur unspecifically without any pattern specific for the resistant or susceptible isolates. In addition, two AA deletions were found for the WR isolate in a non-structurally conserved region. Since one AA change is located in the meander loop and another directly at the end of the Cys-heme binding domain for WR and BSI, the substrate pocket or even the cofactor position inside the core structure could be affected. To investigate the impact of the AA variations on the overall 3D protein structure or ML binding, homology modeling and ML docking for each isolate were conducted.

### 2.3. Homology Models of Haemonchus contortus Cyp-13A11

To examine the impact of AA alterations on the protein structure and potential macrocyclic lactone binding, homology models for *Hco-cyp-13A11* WR, McM, BSI, and ISE (own sequence) were constructed. A tertiary structure homology model was identified using BlastP with the protein data bank (PDB) as database and SwissModel Template Search web tool (https://swissmodel.expasy.org/interactive accessed on 5 February 2022) which identified several human Cyp-3A4 enzyme structures available in PDB. To select the best model for molecular docking studies, only holo-protein crystal structures were further investigated containing the enzyme bound to different ligands or inhibitors expected to allow the docking of MLs. The pre-selected reference structures (PDB: 6MA7, 5A1R, and 4D6Z) were further examined concerning sequence identity towards all *Hco-cyp-13A11* sequences, resolution of the crystal structure, and the structure of the bound inhibitors (Table S3). After performing a multiple sequence alignment using Modeller version 10.1 [51], the crystal structure PDB: 6MA7 was chosen as the template for all homology models. A pairwise alignment was conducted for each *Hco-cyp-13A11* sequence with the template, and 50 homology models were generated for each Modeller run. The homology models were ranked according to their discrete optimized protein energy (DOPE) score. Next, the quality of the homology models with the best DOPE score was assessed with the programs PROCHECK [52], ProSa-web (protein structure analysis) [53,54], and QMEAN (Qualitative Model Energy Analysis) [55,56] to execute individual model refinement.

The Ramachandran plot analysis showed that approximately 99% of all residues were within the generously allowed regions for all homology models (Table S4). The residues corresponding to the outlier regions and possessing sterically disallowed conformations refer to non-conserved loops and could not be optimized via individual loop refinement. However, no major difference between the quality scores of the four homology models was observed.

**Figure 4:**
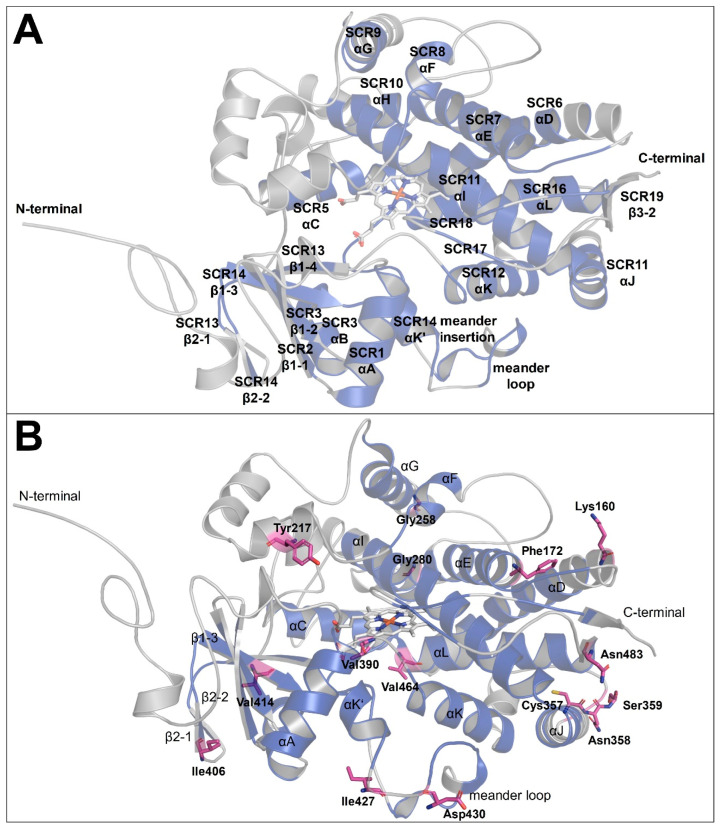
Homology model of Hco-Cyp-13A11. (**A**) Ribbon representation of the molecular model of *Haemonchus contortus* Cyp-13A11/12 ISE isolate. The model was created using Modeller and the human Cyp-3A4 (PDB: 6MA7) as a template. The protein shows the conserved overall fold of Cyps, with a large helical domain and a smaller beta-sheet region. The structurally conserved regions (SCRs) are highlighted in blue, whereas the variable regions are shown in gray. (**B**) The overview of the ISE Hco-Cyp-13A11 homology model displays the distribution of the 16 residue positions (pink sticks) varying between the ISE, McM, BSI, and WR isolates.

Generally, all homology models retained the overall protein fold of the Cyp-3A4 template with predominantly α-helices, a small portion of β-sheets (Figure 4A), and a similar solvent accessible area (ISE: 28056 Å^2^; McM: 26790 Å^2^; BSI: 28052 Å^2^; WR: 27721 Å^2^ as calculated with PyMol version 2.0 [57]). All structural elements of the SCRs predicted by multiple sequence alignment analysis could be confirmed for the homology models.

The highest values of local structure similarities with about 65–90% to the target sequence were computed for the structurally conserved regions between CYPs, particularly the heme-binding core. The lowest local similarities values (5–50%) were determined for the N-terminal tail, the BC-loop, the FG-loop, the GH-loop, and HI-loop regions, since the *Hco-cyp-13A11* peptide sequence comprises within these regions more residues than the Cyp-3A4 template (Figure 3). In particular, the HI-loop, directed towards the surface of the protein structure, is characterized by eleven additional residues. This causes different conformations simulated for this region between the four homology models. Furthermore, thirteen extra AA are found at the end of the N-terminal domain compared to the human Cyp-3A4, suggesting a functional relevance as an N-terminal anchor of this region.

A B-factor analysis used to identify the flexibility of side chains for each homology model revealed higher flexibility for these poorly conserved regions in comparison to human Cyp-3A4 (Figure S4). Interestingly, the BC-loop regions for the susceptible isolates ISE and McM (Figure S4A,B) were predicted to be more flexible compared to the resistant BSI and WR isolates (Figure S4C,D), although this region showed no AA variation due to an SNP. The BC-loop region is described as an essential secondary structure within the CYPs influencing substrate specificity and binding [48].

When comparing all models regarding the predicted AA substitutions, variation primarily occurs randomly in domains towards the outer surface (Figure 4B). No SNP resulting in AA variation drastically influenced the overall protein fold between the Hco-Cyp-13A11 sequences.

However, two variable residues are directly located within the heme-core structure and could potentially affect heme-binding (residue 464) or the recognition and binding of potential substrates (residue 390). The position numbers refer to the ISE reference sequence if not stated otherwise. Therefore, the coordination of the heme-cofactor, particularly the heme propionates, was further investigated, as slight changes of the heme cofactor position within the catalytic pocket can affect its size [60]. Regarding position 464 within the highly conserved Cys-loop region, the isolates WR and ISE show a valine, whereas BSI and McM exhibit a methionine equal to the Cyp-3A4 template, potentially enabling it to form a hydrogen (H) bond and reducing the binding area. Nevertheless, the analysis of the heme-binding site for each model revealed no participation of this specific residue in coordinating the heme or influencing the secondary Cys-loop structure. Rather, the superimposition of all homology models and the Cyp-3A4 template structure indicated a highly conserved Cys-loop fold with Cys461 (ISE, McM, BSI) or Cys459 (WR) at the proximal ligand binding site coordinating the iron heme center (Table S5). Hence, the planar heme cofactor is partially located between the αI and αL helices with the propionates directed to the BC-loop and β1-4 sheet.

**Figure 5:**
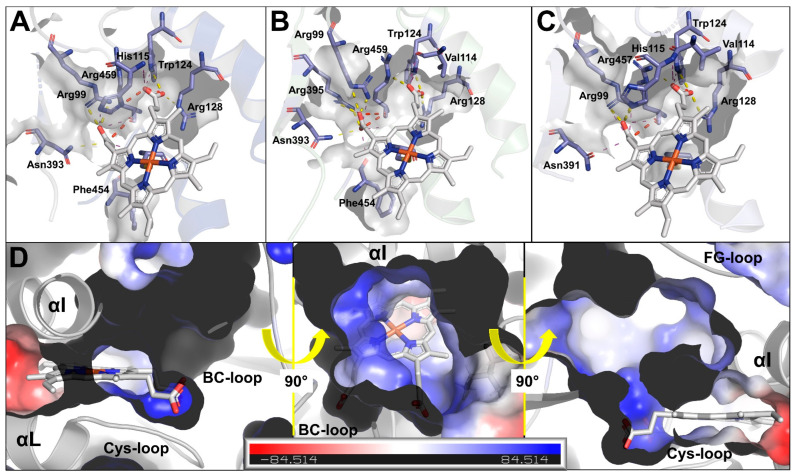
Heme cofactor coordination within Hco-Cyp-13A11 homology models. Coordination of heme propionates and polar interaction sites for Hco-Cyp-13A11 ISE (**A**), McM and BSI (**B**), and WR (**C**) determined with the PyMol Plugin ShowContacts. Hydrogen bonds are presented in yellow, electrostatic clashes in red, and further close contacts (<4 Å) in purple. (**D**) Representative electrostatic surface of the active site cavity. Electrostatic potentials were calculated with PyMOL. Red and blue colors represent the negative and positive electrostatic potentials, respectively.

In addition, slight differences in residues interacting with the heme propionates were observed between all homology models based on PyMol and the Protein-Ligand Interaction Profiler web tool (version 2.2.0) [61] analyses (Table S5). Key residues forming hydrogen bonds with the propionates represent Arg99, Trp124, Arg128, Asn393, Arg395, Arg459 (WR: Arg99, Trp124, Arg128, Asn391, Arg393, and Arg457) (Figure 5). However, one propionate residue is additionally stabilized by a hydrogen-bond, with His115 perpendicularly located above the heme center for ISE and WR isolates (Figure 5A,C). In contrast, no interaction with His115 was computed for the McM and BSI models (Figure 5B). In addition, the heme within the WR model was not predicted to interact with Phe452 as determined for ISE, McM, and BSI (Phe454).

The second variable residue in proximity to the heme-core structure at position 390 (ISE, McM, BSI) is substituted in the WR isolate (Val388Leu) and potentially influences the substrate recognition and/or binding located within the linker region of αK and β1-4. The larger residue side chain directly ranges into the active site pocket but without changing the electrostatics of the cavity. The active site volume for WR with a computed size of 916 Å^3^ was therefore the smallest cavity volume compared to the other homology models (ISE: 1392 Å^3^; McM: 1024 Å^3^; BSI: 1284 Å^3^).

The extent to which the additional methyl group of Leu388 influences substrate recognition remains unclear here. Interestingly, the B-factor analysis of this linker region revealed higher flexibility for the susceptible ISE and McM isolates compared to the resistant BSI and WR (Figure S4). In addition, determination of the electrostatic potential inside the catalytic pocket using PyMol revealed that the pocket surface is primarily hydrophobic or positively charged for all homology models (Figure 5D).

### 2.4. Molecular Modeling of Putative Macrocyclic Lactone-Binding Sites in Hco-Cyp-13A11

To obtain insights into the impact of AA variations towards substrate binding and understanding the observed differences for the MLs in the motility assays, in silico docking was conducted. After generating 3D models of IVM, IVMa, SEL, DRM, EPR, and MOX, the molecules were individually docked into every Hco-Cyp-13A11 homology model using AutoDock Vina (version 1.1.2) [62]. The substrate position with the lowest docking score was selected for iterative energy minimization for each ligand-protein complex. In addition, docking was performed for the Cyp-3A4 template sequence as a reference since the human Cyp-3A4 is known to metabolize IVM [30].

For the human Cyp-3A4, all MLs except EPR docked within the active site (Figure S5) with the highest affinities for IVM (−10.1 kcal/mol) and IVMa (−8.6 kcal/mol). In the case of IVM, the hydroxyl group of the disaccharide moiety was most closely located to the heme iron (distance 3.3 Å). Ivermectin aglycone showed the closest contact with the terminal methyl-group of the alkyl side chain at the spiroketal moiety (distance 4.8 Å). Although SEL, DRM, and MOX also docked within the catalytic pocket, the resulting docking energies were considerably higher (Table 3). Similar to IVM, a hydroxyl group of the DRM disaccharide showed the shortest distance (2.9 Å) to the heme iron. In contrast, the methyl group of the benzofuran substituent of SEL (2.9 Å) and the methyl group of the spiroketal moiety of MOX (5.4 Å) were directed towards the heme center. Apart from SEL and DRM, which both were calculated to interact with Ser119, no substrate formed H-bonds with the active site residues that have been reported in the literature (Tables S5 and S6) [43]. Eprinomectin was predicted to dock at the outer surface of Cyp-3A4 (−8.4 kcal/mol) in a location parallel to the BC-loop and the β-bulge region and lateral of the FG-loop.

**Figure 6:**
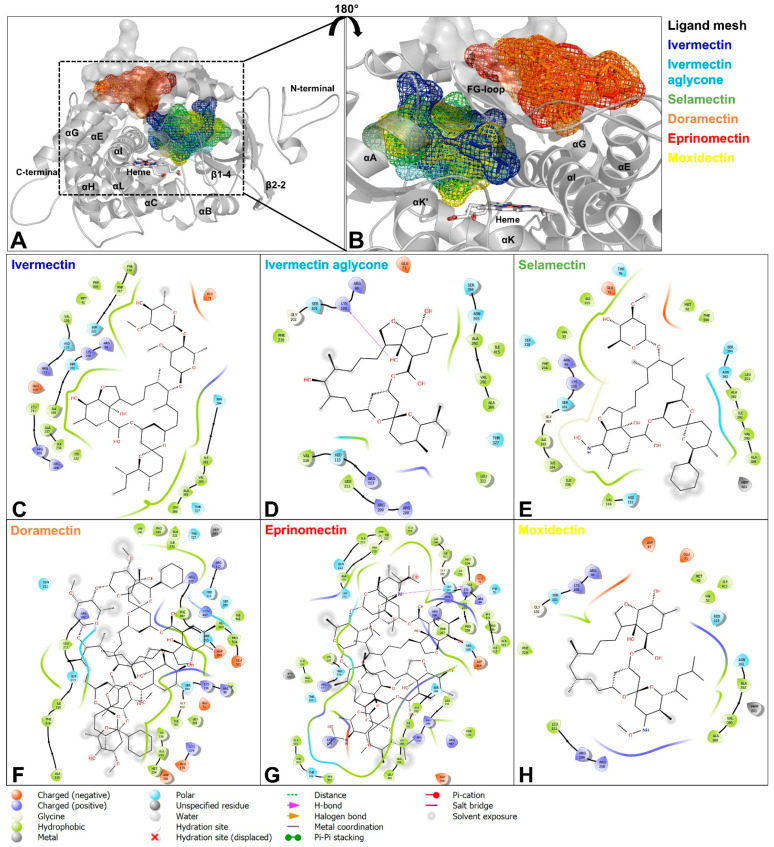
Molecular docking analysis for Hco-Cyp-13A11 Berlin-selected isolate (BSI). (**A**,**B**) Overlayed structures of Hco-Cyp-13A11 BSI in complex with IVM (blue), IVMa (cyan), SEL (green), DRM (orange), EPR (red), and MOX (yellow). The corresponding 2D ML ligand-protein interaction diagrams of Hco-Cyp-13A11 with IVM (**C**), IVMa (**D**), SEL(**E**) and MOX (**H**) show the closest residues within a 4 Å radius. DRM (**F**) docked within a channel leading to the active site. EPR (**G**) showed the lowest docking energy and docked at the outer surface of the BC-loop. 2D interaction plots were generated using Maestro Elements (version 4.6.117).

Calculations for the Hco-Cyp-13A11 models demonstrated considerable differences in docking energies compared to the human Cyp-3A4. IVM, IVMa, SEL, and MOX were predicted to bind with generally lower energies ranging from −5.9 to −11.3 kcal/mol within the active site for all homology models (Table 3, Figure 6, Figures S6–S8). In addition, no considerable differences between the models nor a correlation between differences in models and the resistance status were observed (Table 3). However, the MLs were predicted to have different interaction sites (Table S6) within the four models and orientations within the catalytic pocket for the lowest docking energy positions.

Investigating IVM, the spiroketal moiety was located towards the heme cofactor in the ISE, BSI, and WR homology models. At the same time, the best docking position for McM indicated IVM to be directed towards the center with the benzofuran substituent. The analysis of IVMa predicted that this molecule could interact with the heme center via the alkyl side chain of the spiroketal moiety while it gets potentially metabolized at the benzofuran by the WR Hco-Cyp-13A11. In the case of SEL, the molecule was directed with the sugar substituent to the catalytic center of McM and WR while it was flipped within BSI and ISE. There, SEL potentially interacts via the cyclohexyl side chain with the heme center. Moxidectin showed the fewest commonalities regarding the predicted binding topology between the homology models. Docking for WR and McM presented the methoxime moiety above the heme iron. For the BSI model, MOX most likely interacts with the methyl of the spiroketal group. In contrast, MOX was directed with the benzofuran group to the catalytic site for the ISE model. For both EPR and DRM, docking into the active site was not feasible for the WR model. The best-fit position was predicted to occur on the protein surface. Both molecules were simulated to bind to the FG-loop’s peripheral site, a highly hydrophobic region that is predicted to reside within the lipid bilayer and the *N*-terminal membrane anchor α-helix [63].

The same binding locations (Figure S8) and docking energies (Table 3) for EPR and DRM were identified for the BSI Hco-Cyp-13A11 model. While the DRM binding site for both WR and BSI models shared Arg209 and Arg497 (WR: Arg495) as interacting residues, EPR showed no conformity (Figure 6, Figure S8, Table S6). In contrast to the WR and BSI models, docking simulations for the ISE and McM models resulted in EPR and DRM binding within the active site (Figures S6 and S7). Both models for the susceptible isolates already predicted higher cavity volumes than the resistant isolate models.

## 3. Discussion

Cyps have already been reported to contribute to resistance in cancer (Rochat, 2005; Rodriguez-Antona and Ingelman-Sundberg, 2006) and against insecticides [64,65]. Even though the primary resistance mechanism against the broad-spectrum anthelmintics benzimidazoles (BZs) is linked to SNPs of the beta-tubulin gene of nematodes [66,67,68], an increased oxidation level of different BZs was observed in *C. elegans*, *H. contortus,* and *Fasciola hepatica* [69,70,71,72,73].

In *C. elegans*, the ablation of cytochrome P450 activity with the widely used Cyp inhibitor piperonyl butoxide or the use of a severely impaired strain in cytochrome reductase activity resulted in slightly increased susceptibility to IVM but not MOX [74]. The authors further excluded that Cyp-14A5 contributes to changes in IVM susceptibility, although it was previously shown to be moderately upregulated in IVM and MOX-selected *C. elegans* strains [20]. The present study aimed to investigate the impact of the transgenically expressed *Hco-cyp-13A11* in the intestine of *C. elegans* regarding the susceptibility of different MLs.

Using extrachromosomal arrays to express transgenes represents a fast and efficient technique for analyzing gene functions. Within this study, microinjection was performed into the *C. elegans* N2 background since *C. elegans Cel-cyp-13A11* and *Cel-cyp-13A12* loss-of-function strains were unavailable to perform rescue experiments. Various studies have already demonstrated the suitability of transgene expression to investigate drug targets against parasitic nematodes [15,39,75,76,77].

The analyzed *Hco-cyp-13A11* was derived from the highly resistant White River isolate and its expression in the gut epithelium has decreased susceptibility for IVM, IVMa, and SEL. In contrast, no effect was observed for EPR, DRM, and MOX compared to the N2 wild-type control line. This study demonstrates for the first time that ML sensitivity in *C. elegans* can be modulated by transgenic expression of a parasitic nematode Cyp.

Interestingly, neither a constitutive expression nor a direct link to the modulation of ML susceptibility or xenobiotic inducibility of *Hco-cyp-13A11* has been reported so far. The introgression of two highly resistant *H. contortus* isolates into a susceptible background identified a quantitative trait locus (QTL) for IVM resistance. This QTL was localized on chromosome V ranging from 37 to 42 Mbp, and the same region was under selection using two geographically and genetically divergent IVM resistant populations [78]. Although *Hco-cyp-13A11* is located on chromosome V (area of 14.2 Mbp) in proximity to the QTL region (37-42 Mbp), it seems not to be part of the QTL under selection. However, there are several possible mechanisms by which *Hco-cyp-13A11* might nevertheless be involved in modulating the susceptibility of worms to MLs. Due to the high genomic variability of *H. contortus*, different genomic regions might be involved in other isolates and it might be difficult to obtain statistical support for regions with only a small effect. The major QTL regions might be under selection but exert its effects through effectors encoded in other regions that might include *Hco-cyp-13A11*. Alternate splicing events have not been considered here but might lead to changes in the protein structure, augmenting the gene function or translation efficacy as reported for *C. elegans* [79] and potentially increasing the resistance level. For example, *Brugia malayi* revealed a sex-dependent differential splicing of the potassium channel *slo-1* causing significant differences in emodepside sensitivity between females and males [80]. Moreover, alternate splicing of the acetylcholine receptor subunit *acr-8* resulted in truncated transcripts that were found to be expressed explicitly in levamisole resistant *H. contortus*, *Trichostrongylus colubriformis,* and *T. circumcinta* [81,82].

Investigations on the orthologues *C. elegans* Cyp *cyp-13A12* co-expressed with the Cyp reductase *emb-8* in insect cells revealed its role as an epoxygenase of polyunsaturated fatty acids [83]. Although further functional information and tissue expression levels are missing, the miscellaneous role of this particular *C. elegans* Cyp does not exclude the hypothesis that *Cel-cyp-13A12* might also contribute to ML resistance. This should be investigated in the future by creating loss-of-function alleles and examining the effects of overexpression of *Cel-cyp-13A12* but also investigating its closely related paralog *Cel-cyp-13A11*.

Within this study, the transgenic expression of *Hco-cyp-13A11* had the most potent effect for IVM with a 4.3-fold increase of the EC_50_ value, followed by SEL with 2.6-fold and a modest increase for IVMa with a 1.7-fold change. Different studies have compared the interaction of IVM and MOX with recombinantly expressed Pgps, and in all cases, the interaction of IVM was much stronger than for MOX [1,84,85]. However, only one study compares the effects of a similar spectrum of MLs on Pgps. Kaschny et al. [86] expressed *Cylicocylus elongatus pgp-9* in *Saccharomyces cerevisiae* yeast cells. This data revealed a strong interaction with IVM and EPR, a moderate interaction with MOX, and no interaction with SEL and DRM [86]. Ivermectin aglycone was not tested in this study. Although the different order of effect strength for the different MLs shows specific interactions with the particular Pgp, the fact that IVM always showed the strongest effects is remarkable. However, this needs more diverse studies since most investigations only compared IVM to MOX [39,84,85].

Except for the milbemycin MOX, all tested drugs belonged to the class of avermectins differing in the sugar moiety, the alkyl side chain, a methoxime function, or a functionalized benzofuran. In particular, the sugar group was shown to strongly affect the physico-chemical properties of these drugs, for example, the hydrophobicity results in different logP values [1]. This further influences pharmacokinetics and pharmacodynamics, mainly demonstrated for IVM and MOX, potentially explaining the observed differences for IVM, SEL, and IVMa [6,20,85,87,88]. Ivermectin and MOX were shown to bind to the same glutamate-gated chloride channel (GluCl) subunit, but with different affinities towards the binding site [87]. The analysis of the GluCl crystal structure reinforced the previous observations made for IVM interactions and proposed a model for the IVM binding site. The missing disaccharide and the additional methoxime moiety for MOX suggest different interactions for MOX within this structure [89]. A difference between IVM and MOX can also be assumed for ML metabolism by *H. contortus*. While MOX was reported to be metabolized [33], no IVM metabolites have been identified [34]. Since the latter study by Vokral et al. (2013) used a susceptible *H. contortus* isolate to analyze metabolism, the production of IVM metabolites cannot be hypothesized for resistant isolates containing SNPs or overexpressing xenobiotic metabolizing enzymes.

The transgenic expression of *Hco-cyp-13A11* did not affect the susceptibility to MOX, EPR, and DRM, thereby confirming the fact that there are considerable pharmacokinetic differences between different MLs. However, the sugar moiety does not seem to be the pivotal functional group in deciding whether a ML can interact with *Hco-cyp-13A11*, since IVM, EPR, and DRM all possess a disaccharide group in contrast to MOX, but *Hco-cyp-13A11* expression was only protective against IVM. Further structural differences are present at the alkyl side chain of MOX and an additional carbon-carbon double bond at the spiroketal moiety of EPR and DRM.

To better understand why *Hco-cyp-13A11* shows a substrate specificity towards the tested MLs, comparison in the observed differences in EC_50_ values, molecular modeling and docking studies were performed. Although analysis of *Hco-cyp-13A11* in *C. elegans* was carried out only for the sequence variant derived from the White River isolate, in silico analyses were extended to *Hco-cyp-13A11* sequences obtained from three additional isolates differing in resistance status. Human Cyps have already been shown to exhibit polymorphisms in multiple allelic variants associated with differences in metabolizing drugs [88,90]. In total, the exon sequences of *Hco-cyp-13A11* from four *H. contortus* isolates revealed 14 polymorphic positions in the amino acid sequences. However, none of these polymorphisms occurred explicitly in the ML-susceptible or -resistant isolates. This can certainly be linked to the high genetic diversity of *H. contortus* with a mutation rate ten times higher than in vertebrates, resulting in considerable variation within a laboratory strain as observed for the sequences derived from the partially inbred ISE [42,91,92]. Additional different selection pressures can affect the genes selected and how they respond to MLs, thereby leading to different phenotypes within different ML-resistant strains [93,94].

Nonetheless, two AA changes were predicted in SCRs directly located towards the active site of the Cyp and potentially influencing substrate binding. Molecular modeling of Hco-Cyp-13A11 calculated that the other AA variations occurred mainly towards the protein’s surface. This may lead to slight changes in electrostatic interactions with membrane lipids, affecting Cyp behavior as proposed for human Cyps [95]. The more positively charged AA are located on the protein’s surface, the stronger it is attracted to anionic membranes compared to neutral ones therefore changing the orientation of Cyp towards the membrane and its immersion depth in the membrane. Hence the flexibility in the membrane-immersed parts of the catalytic domain such as the FG-loop and reduction in access for substrates to their channels [95]. However, molecular dynamic simulations should be conducted to evaluate the impact of all AA changes on the substrate access channel gating.

In general, the molecular models for Hco-Cyp-13A11 revealed high sequence identities for the helices I, L, J, K, and the two sets of beta-sheets compared to the human Cyp-3A4. Furthermore, the ExxR and the CIG sequence motifs which are known to stabilize the core, and the heme-binding are also preserved for Hco-Cyp-13A11 [48]. This core structure was shown to be highly conserved in Cyps of all phyla [48,96]. Although the primary sequence forming the helices E and D showed a low sequence coverage for Hco-Cyp-13A11 among the isolates, which were predicted to be well-conserved core structures in mammals [49], the secondary helix structure was preserved.

Interestingly, Hco-Cyp-13A11 exhibits more residues within the G helix (6 AA) and the HI-loop (11 AA) than Cyp-3A4. Even though the exact function of the HI-loop at the surface of the protein remains unclear, the quantity of AA within Hco-Cyp-13A11 can affect the opening and closing of the substrate access channels through altered flexibility and conformational dynamic [64,97].

The short meander insertion region of Hco-Cyp-13A11 indicates that this protein can be potentially classified as a class II Cyp. The classification refers to the electron transfer mechanism to the catalytic site with class II Cyps only requiring a flavin adenine nucleotide containing P450 reductase to transfer electrons. In contrast to class II, class I Cyps require a reductase and an iron-sulfur redoxin, while class III Cyps do not require any electron donor [50]. Together with αJ/αJ’, the meander insertion region is proposed to display a reductase interaction face [51]. Keller et al. (2014) has already revealed the importance for the orthologous *Cel-cyp-13A12* to be co-expressed with the reductase *emb-8* to build a monooxygenase system.

The low sequence identity for the non-core regions between human Cyp-3A4 and Hco-Cyp-13A11 is not surprising. Homologous Cyps are not necessarily expected to have the same spectrum of specificity or affinity. It can be assumed that the nematode Cyp metabolizes a particular drug that is no substrate to Cyp-3A4 and the other way around. 

Human Cyp-3A4 metabolizes several large substrates, including erythromycin and cyclosporin [23,98]. The present study indicated favorable docking scores for the MLs IVM, IVMa, SEL, DRM, EPR, and MOX when in silico complexed with the human Cyp-3A4 crystal structure (PDB: 6MA7). Except for EPR, all drugs could dock to the active site cavity within the protein. Cyp-3A4 has already been associated with cross-resistance to several drugs [23]. Independent biotransformation studies for IVM [30] and MOX [32] conducted with hepatic microsomes described the ability of the Cyp-3A family and particular Cyp-3A4 to metabolize these drugs. The best IVM docking score within this study was accomplished with the disaccharide moiety directed to the heme center. Indeed, IVM metabolite analysis showed a demethylation product at the sugar group [30,31]. The in silico calculations for MOX exhibited the closest contact towards the heme with the methyl-group positioned at the spiroketal function, and a corresponding hydroxylation product has also been identified [99,100]. However, these metabolite formation studies pointed out more than one metabolization site for IVM and MOX.

Multiple docking modes within Cyp-3A4 were also reported for erythromycin and ketoconazole and depended on the binding of an effector molecule and the molecular size of the substrate [101]. It should be noted that the analysis in the present study focused only on the best docking score for each drug. Ivermectin, MOX, IVMa, and SEL were docked with different conformations into the Cyp-3A4 active site exhibiting differing docking scores. Therefore, it can be assumed that Cyp-3A4 can also metabolize IVMa, SEL, and DRM. In contrast to the other MLs, EPR showed the highest docking score on the solvent-accessible site between the BC-loop and the beta bulge region. The BS-loop represents one of the most flexible parts of the protein, influencing the formation of substrate access channels to bind drugs selectively [95,97]. Thus, it currently cannot be excluded that EPR cannot bind within the active site.

The Hco-Cyp-13A11 models generated here suggested that the protein can form complexes with each ML, but docking scores for SEL, DRM, and MOX were considerably higher than those obtained for human Cyp-3A4. For Hco-Cyp-13A11, no major differences regarding drug docking scores or drug interacting residues could be observed between the models for predicted sequences from susceptible and resistant isolates. Analyzing predicted hydrogen-bond formation with MLs in the active site, Lys100, Arg113, and Ser394 were identified as potential hot-spot residues for substrate recognition in all Hco-Cyp-13A11 models. Interestingly, the models for WR and BSI showed docking of DRM and EPR at the solvent-accessible site of the FG-loop. The observed peripheral docking area has already been described for the human Cyp-3A4 when exposed to the highly hydrophobic bromoergocryptine and ritonavir. It is assumed that these molecules first bind outside and are then translocated to the catalytic cavity due to conformational changes [64]. Currently, it cannot be excluded that Hco-Cyp-13A11 WR and BSI would also allow translocation of EPR and DRM to the active site.

In contrast, the active site of ISE and McM models was sufficient to complex both molecules. It remains unclear if the smaller active sites of the WR and BSI models, presumably caused by the long-distance effects of polymorphisms at positions outside of the active site, are reliable or caused by artifacts in the modeling process.

The FG-loop, which has been reported to be immersed into the membrane in many Cyps, represents, similar to the BC-loop, one of the most flexible regions in Cyps and is capable of opening and closing substrate access channels [95]. Nevertheless, the amphiphilic character of EPR and DRM would enable them to enter the active site from the membrane environment, which cannot be excluded since channel formation is dependent on different environmental factors such as membrane composition or cholesterol concentration within the membrane [23,95].

Although no IVM biotransformation products for the susceptible ISE isolate were detected [34], the importance of Cyps increasing the ML resistance should not be underestimated. This is mainly because MOX oxidation products were identified for *H. contortus* [33], and the docking study revealed the ability of multiple MLs to bind to *Hco-cyp-13A11*.

However, the identified SNPs leading to 14 amino acid changes within *Hco-cyp-13A11* between the analyzed isolates ISE, McM, WR, and BSI do not seem to contribute to specific responses of resistant and susceptible isolates towards MLs. It needs to be investigated if *Hco-cyp-13A11* might be differentially expressed upon ML exposure as already shown for HCON_00038960 [19]. *Hco-cyp-13A11* could be one among several players leading to ML resistance, as several studies already support a multigenic basis for ML resistance [42,78,102,103].

Besides Cyps, flavin-containing monooxygenases, glutathione-*S*-transferases or glycosyltransferases contribute to drug metabolism in humans, ruminants, and nematodes by being upregulated or reinforcing the metabolic cascade [13]. Particularly, members of the drug transporter family P-glycoproteins such as *H. contortus* and *Parascaris univalens pgp-2* [17,104] *Cylicocyclus elongatus*, *H. contortus* and *Parascaris univalens pgp-9* [86,104,105], *T. circumcincta pgp-10* [16], *Parascaris univalens* and *H. contortus pgp-11* [15,19], and *Hco-pgp-13* [106], were linked to IVM resistance. A structural model analysis of *Hco-pgp-13* additionally predicted a high-affinity binding site in the inner chamber of the protein for IVM [106]. Similarly, high binding affinities were also calculated for the *Cel-pgp-1*, which was also predicted to bind EPR, DOR, SEL, MOX, IVMa, and abamectin within the same cavity [107]. P-glycoproteins and cytochrome P450 enzymes, especially the human Cyp-3A family, possess broad substrate specificities and were shown to overlap in substrate and inhibitor specificity [23,108,109]. Both human Pgps and Cyps are frequently expressed in the same cells, such as hepatocytes and enterocytes, demonstrating their functional link within the drug detoxification cascade [23]. Hence, Pgps can alter the intracellular concentration of Cyp inducers or substrates and result in changing the magnitude of inductive and catalytic response. Though detailed data about the tissue-specific expression of Pgps and Cyps in *H. contortus* are missing, similar coherent expression patterns can be deduced from different studies. Both *Hco-pgp-13* [106], *Hco-pgp-2* [17], and *Hco-cyp-34A5*, *Hco-cyp-13A10*, *Hco-cyp-33C9*, *Hco-cyp-43A1* [24] show higher expression levels in the intestine of adult *H. contortus* than in other tissues. However, inducibility upon IVM exposure was only shown for *Hco-pgp-13* so far [19].

In conclusion, this is the first report of a transgenically expressed parasitic nematode Cyp decreasing IVM, IVMa, and SEL susceptibility. However, molecular docking studies could not verify substrate specificity or resistance-specific mutations for *Hco-cyp-13A11* resulting in different affinities towards the MLs. Therefore, *Hco-cyp-13A11* is assumed to contribute to ML resistance in *H. contortus* only when combined with other pathways.

## 4. Materials and Methods

### 4.1. Chemicals

Stock solutions of 10 µM IVM (Sigma-Aldrich, I8898, Taufkirchen, Germany), 10 µM MOX (Sigma-Aldrich, 33746), 100 µM IVMa (Santa Cruz, SC-202189, Heidelberg, Germany), 10 µM EPR (Cayman, 28182, Ann Arbor, MI, USA), 100 µM SEL (Sigma-Aldrich, 32476, Darmstadt, Germany) and 10 µM DRM (Sigma-Aldrich, 33993, Germany) were prepared in 100% DMSO. For thrashing assays with the *C. elegans* N2 Bristol and transgenic strain expressing Hco-cyp-13A11, stock solutions were serially diluted to obtain final concentrations ranging from 0.1 to 1 µM IVM, 0.25 to 2.5 µM MOX, 10 to 100 µM IVMa, 0.05 to 2 µM EPR, 20 to 200 µM SEL, and 0.5 to 2 µM DRM.

### 4.2. Plasmid Construction for Transgenesis

For transgenesis, a plasmid containing the 1551 bp *H. contortus* White River (WR) *cyp-13A11* (old designation: HCOI00827700, now: HCON_00141052, BioProject PRJEB506) placed downstream of a 2000 bp *C. elegans ges-1* promoter fragment followed by a FlagTag and 822 bp of the *C. elegans* 3′-UTR of the *unc-54* gene was constructed (Figure S1). To amplify *ges-1* and the *unc-54* 3′-UTR, genomic DNA was isolated from *C. elegans* Bristol N2 using NucleoSpin Tissue XS kit (Macherey Nagel) according to the manufacturer’s instructions. To amplify *Hco-cyp-13A11*, RNA was isolated from fourth-stage larvae and cDNA synthesized as described previously [28]. Each fragment needed to assemble an expression plasmid was amplified via PCR or RT-PCR. Corresponding primer sequences were designed using the NEBuild-er^®^Assembly tool (version 2.3.1). Reactions contained 0.02 U/µL Phusion Hot Start II High-Fidelity DNA polymerase (Thermo Scientific, Waltham, MA, USA), 0.2 µM dNTPs, 0.5 µM of each primer in 50 µL 1 × Phusion HF buffer. Reactions were denatured at 98 °C for 2 min, followed by 50 cycles of 98 °C for 5 s, a primer-pair specific annealing temperature for 30 s, and 72 °C for 2 min. Primer sequences and specific annealing temperatures are provided in the supporting data (Supplementay Table S1). The purified PCR products (DNA Clean & Concentrator™-5, Macherey Nagel) were used for assembly with the NEBuilder^®^ HiFi DNA Assembly Master Mix and were cloned with NEBuilder HiFi DNA Assembly Cloning Kit (New England Biolabs) according to the manufacturer’s instructions. The pUC19 vector (New England Biolabs) was linearized with SmaI (Thermo Scientific) before assembly. The resulting plasmid was confirmed by Sanger sequencing (LGC Genomics, Berlin, Germany).

### 4.3. Transformation of Caenorhabitis elegans

The *C. elegans* Bristol N2 strain obtained from *Caenorhabditis* Genetics Centre (CGC, University of Minnesota, Minneapolis, MN, USA) was used as wild-type and maintained under standard conditions [110]. The plasmid for the expression of *H. contortus* cyp-13A11 was diluted in water and injected into the germline of young adult *C. elegans* hermaphrodites at a concentration of 50 ng/μL as described previously [39,75]. A plasmid carrying a pharyngeal GFP-expression marker (pPD118.33, Addgene plasmid 1596: L3790, P_myo2_-*gfp*, Fire Lab 1995 Vector Kit) was co-injected as a transformation marker at a concentration of 12.5 ng/μL. Successfully transformed worms were identified in the F1 progeny of injected worms by GFP fluorescence and isolated on new agar plates (Figure S2). RT-PCR confirmed the expression of *Hco-cyp-13A11*. For this purpose, GFP positive transgenes, all descending from the same F1 worm, were collected, homogenized with a pestle, and RNA isolated with the NucleoSpin RNA kit (Macherey Nagel) according to the manufacturer’s instructions. Then, cDNA was synthesized from approximately 1 µg of RNA using the Maxima first-strand cDNA synthesis kit (Thermo Fisher) according to the manufacturer’s instructions. PCR was conducted in a 50 µL reaction mixture containing 2 µL cDNA or no-RT control template, 0.02 U/µL Phusion Hot Start II High Fidelity DNA polymerase (Thermo Scientific), 0.2 mM dNTPs, 0.25 µM of each gene-specific expression plasmid primer (Table S1) in 50 µL 1 × Phusion HF buffer. The reaction was denatured at 98 °C for 2 min, followed by 45 cycles of 98 °C for 15 s, annealing at 57.8 °C for 30 s and 72 °C for 2 min. The presence of the *Hco-cyp-13A11* PCR product was verified by gel electrophoresis and Sanger sequencing.

### 4.4. Synchronization of Caenorhabditis elegans Developmental Stages

For synchronization, 100 µL of pelleted worms were exposed to 2 mL of a lysis solution consisting of distilled water, 1 M NaOH, and chlorine-based household bleach in a 1:5:4 ratio. The obtained eggs were washed four times with a 15 mL M9 buffer (3 mg/mL KH_2_PO_4_, 6 mg/mL Na_2_HPO_4_, 5 mg/mL NaCl, 1 mM MgSO_4_) and centrifuged for 2.5 min at 780 rcf. Finally, the eggs were resuspended in 2 mL M9 buffer, placed on nematode growth medium (NGM) agar plates seeded with *Escherichia coli* OP50 (Brenner, 1974), and left to hatch at 20 °C.

### 4.5. Motility Assays

A minimum of 12 young adult transgenic individuals in 100 µL M9 buffer were added to 1880 µL S Medium (prepared by mixing 1 l S Basal (5.85 mg/mL NaCl, 6 mg/mL K_2_HPO_4_, 0.005 mg/mL cholesterol), 10 ml trace metal solution (1.86 mg/mL disodium EDTA, 0.69 mg/mL FeSO_4_∙7H_2_O, 0.2 mg/mL MnCl_2_∙4H_2_O, 0.29 mg/mL ZnSO_4_∙7H_2_O, 0.025 mg/mL CuSO_4_), 3 ml 1M CaCl_2_ and 3 mL 1M MgSO_4_) containing *E. coli* OP50 (OD600 approximately 0.5) and 20 µL drug solution per concentration into a 6 well plate. DMSO was used as vehicle control and accounted for 1% of the total volume of each well. Assays were run, protected from light, at 20 °C with constant shaking at 150 rpm for 24 h. The worms were then transferred to an NGM agar plate overlaid with M9 buffer and allowed to adapt to light for 1 min before the movement was quantified under a stereo microscope by counting the number of body bends for 1 min. Each concentration was repeated at least four times independently with three worms per block (n ≥ 12 worms for each concentration). To compare the thrashes per minute of the N2 control strain and the transgenic *Hco-cyp-13A11 C. elegans* strain for the DMSO control, a Whitney Mann *U* test was used (Figure S3) (GraphPad Prism 5.0.3). For the concentration-response curves, the mean number of movements was normalized to the mean of the no-drug control of the same strain in the same block to obtain the relative motility as a percentage. For concentration-response curves, EC50 values were determined by four-parameter logistic regression using GraphPad Prism 5.0.3, and statistical differences in EC50 values were calculated using the extra sum of the squares F test. The parameters’ top and bottom were constrained to values between 0 and 100%.

### 4.6. Parasite Isolates

Four isolates of *H. contortus* with differing susceptibility to MLs and other anthelmintics were used for *Hco-cyp-13A11* (HCON_00141052) sequence comparison.

(i) McM (McMaster): susceptible to all anthelmintics.
(ii) ISE (Inbred-susceptible Edinburgh; MHco3): susceptible to all anthelmintics.
(iii) BSI (Berlin selected isolate): highly IVM, MOX, thiabendazole (TBZ), and levamisole (LEV) resistant.
(iv) WR (White River; MHco4): highly IVM and BZ resistant; moderately LEV resistant.

All isolates have been maintained by the regular passage in helminth-naïve lambs at the Institute for Parasitology and Tropical Veterinary Medicine of the Freie Universität in Berlin for several years. The WR isolate was regularly challenged by treating the infected animals with ivermectin and fenbendazole.

### 4.7. Sequence Comparison of Hco-cyp-13A11 from Different Haemonchus contortus Isolates

According to the manufacturer’s instructions, DNA was isolated from infective third-stage larvae using the NucleoSpin Tissue kit (Macherey Nagel). To amplify exons encoding the CYP gene, primer pairs were designed at the 5′- and 3′-ends of either single exon sequences or approximately over-spanning three exon sequences (Table S2). All PCRs were conducted using Phusion Hot Start II High Fidelity DNA polymerase (Thermo Scientific). Reactions contained 0.02 U/µL polymerase, 0.2 mM dNTPs, 0.25 µM of each primer in 20 µL 1 × Phusion HF buffer. Reactions were denatured at 98 °C for 2 min, followed by 42 cycles of 98 °C for 20 s, a primer-pair specific annealing temperature (Table S2) for 30 s, and 72 °C for 1 min. Amplification products were cloned into the pSC-B-amp/kan vector (StrataClone Blunt Cloning Kit, Agilent Technologies). Plasmids (GenUP™Plasmid Kit, biotechrabbit) with inserts of the expected size were identified by restriction analysis, and clones with such inserts were sequenced by primer walking at LGC Genomics (Berlin). Exon sequences encoding *Hco-cyp-13A11* have been deposited in the GenBank database under the accession numbers OM99853 (ISE), OM995854 (McM), OM995855 (WR), OM995856 (BSI).

### 4.8. Initial Analysis of DNA and Deduced Protein Sequences

The multiple sequence alignments were obtained by Clustal Omega version 1.2.4 (Available online: https://www.ebi.ac.uk/Tools/msa/clustalo/ accessed on 12 February 2021). The translation of *Hco-cyp-13A11* protein-coding genomic DNA fragments into a polypeptide sequences was performed using the ExPASy–Translate tool (Available online: https://web.expasy.org/translate/ accessed on 18 Februry 2021). The protein parameters (molecular weight, length, theoretical isoelectric point (pI)) were calculated with ExPASy–ProtParam (Available online:https://web.expasy.org/protparam/ accessed on 19 February 2021) and ExPASy–ScanProsite (Available online: https://prosite.expasy.org/scanprosite/ accessed on 19 February 2021) was used to detect functional motifs in the sequence. The prediction of secondary structure elements was carried out by the web tool ESPript version 3.0 (Available online: https://espript.ibcp.fr/ESPript/cgi-bin/ESPript.cgi accessed on 19 February 2021) [59] using the crystal structure of the human Cyp-3A4 (PDB: 1TQN) as a structure prediction reference [43].

### 4.9. Haemonchus contortus Cyp-13A11 Homology Modeling

Based on the crystal structure of *Homo sapiens* Cyp-3A4 (PDB: 6MA7) [111] as a template, 3D models of Hco-Cyp-13A11 were built using Modeller version 10.1 [51]. After pairwise alignment of each Hco-Cyp-13A11 isolate sequence with the Cyp-3A4 target sequence, 50 homology models were calculated in each Modeller run. The homology models were then individually improved based on the Modeller loop refining method by calculating 50 loop-refined models to decrease the number of Ramachandran-plot outlier residues. Subsequently, model qualities were assessed by DOPE score, the QMEAN (qualitative Model Energy Analysis) scoring function [55,56], ProSA-web (Protein structure analysis) [53,54], and Errat [112]. Next, an iron-oxo haem molecule was included by identifying the best docking position with AutoDock Vina version 1.1.2 [63]. Therefore, the homology models were prepared using AutoDock 4 [113] by adding polar hydrogens and Kollman charges. Furthermore, the heme ligand was processed within AutoDock 4 by keeping the structure rigid and adding polar hydrogens and Gasteiger charges. The grid box used to dock the heme ligand was built manually within AutoDock 4 by centering the box to the iron coordinating cysteine sulfur (ISE, McM, BSI: Cys461; WR: Cys459). The lowest energy position calculated by AutoDock Vina was selected for each model, and interacting residues within the protein were analyzed using PyMOL version 2.0 [57]. Finally, the iron-oxo heme was connected to the homology model by creating a covalent bond between the heme iron and the sulfur atom of the previously mentioned cysteine residues using PyMOL. Heme cofactor coordinating residues were analyzed using the Pimolin plugin ShowContacts and the PLIP Protein-Ligand Interaction Profiler web tool version 2.2.0 [62]. The determination of the active site volume was performed with the web tool CASTp [114] (version 3.0, probe radius 1.4 Å).

### 4.10. Molecular Docking of Macrocyclic lactones into the Hco-Cyp-13A11 Homology Models

AutoDock Vina was used in all docking experiments with the homology models described previously. The analyses performed were restricted to molecular docking without performing an additional molecular dynamics simulation. Before docking, the heme-containing receptor was prepared with AutoDock Tools by adding polar hydrogens and Kollman charges. Thereby, AutoDock Tools automatically set the heme iron charge to zero. In addition, water molecules present in the protein structure were removed.

The molecular structures of the MLs used for ligand docking were all downloaded from PubChem (PubChem CID: IVM (6321424); IVMa (126455999); SEL (9578507); DRM (9832750); EPR (6450531); and MOX (9832912)). In the case of IVM and EPR with occurring mixtures of B1a and B1b, only the structure of the predominant variant B1a was used for docking simulations. The 3D structures used for docking were generated with ChemDraw3D (version 20.0.0.41, level: ultra), and energy conformation was minimized performing the MMF94 (Merck molecular force field) approach. The ligands were then processed within AutoDock 4 by keeping the structures flexible and adding polar hydrogens and Kollman charges.

All MLs were primarily docked to the active site cavity of the models, which were identified using the PyMol APBS (Adaptive Poisson-Boltzmann Solver) [115] plugin calculating the macromolecular electrostatics and the CAVER3 [116]plugin computing tunnels and channels in protein structures. In the next step, the initial grid box (40 Å × 40 Å × 40 Å) used for each ligand docking was centered on the heme cofactor of each homology model. In the AutoDock Vina configuration files, the parameter *num_modes* was set to 100, the *exhaustiveness* to 12, and *energy range* to 9. Based on the lowest docking energy resulting from the calculation and visual evaluation with PyMol, grid boxes were iteratively improved, and analysis with the same configuration files was performed again until obtaining the lowest (most negative) docking energies possible. If docking did not result in binding to the active site, the best docking position on the molecular surface was screened. The evaluation of interacting residues was performed by PyMol, the open-source PLIP Protein-Ligand Interaction Profiler web tool [62], and the ligand interaction tool of Maestro Elements version 4.6.117. The latter tool was also used to generate 2D ligand-protein interaction diagrams.

## Figures and Tables

**Table 1:** Effect of *Hco-cyp-13A11* transgenic expression on macrocyclic lactone susceptibility in *Caenorhabditis elegans* N2 background recorded by counting individual motility (body bends in liquid medium).

Strain	Drug	EC_50_ (95% CI ^1^) [nM]	R^2 2^	*p* Value	Fold Change in EC_50_ vs N2
N2 ^3^	Ivermectin	1.64 (1.43–1.87)	0.972	<0.0001	4.27
*Hco-cyp-13A11* ^4^	Ivermectin	7.01 (5.53–8.89)	0.874		
N2	Ivermectin aglycone	163.6 (152.6–175.4)	0.984	<0.0001	1.68
*Hco-cyp-13A11*	Ivermectin aglycone	275.5 (259.7–292.2)	0.980		
N2	Selamectin	156.7 (136.8–179.5)	0.983	<0.0001	2.58
*Hco-cyp-13A11*	Selamectin	405.3 (386.1–425.3)	0.950		
N2	Doramectin	7.75 (7.30–8.24)	0.987	0.0005	1.10
*Hco-cyp-13A11*	Doramectin	8.60 (8.11–9.12)	0.986		
N2	Eprinomectin	1.48 (1.17–1.86)	0.994	0.1165	1.27
*Hco-cyp-13A11*	Eprinomectin	1.89 (1.39–2.56)	0.972		
N2	Moxidectin	5.95 (5.38–6.58)	0.966	0.3212	1.07
*Hco-cyp-13A11*	Moxidectin	6.40 (5.81–7.05)	0.955		

^1^ 95% confidence interval. ^2^ Coefficient of determination. ^3^ Genotype: wild-type. ^4^ Genotype: gutCyp-13A11/12Ex1 [Cel-ges-1p::Hco-cyp-13A11::FLAG::Cel-unc-54_3′-UTR; Cel-myo-2p::gfp::Cel-unc-54_3′UTR].

**Table 2:** Prediction of Hco-Cyp-13A11 protein parameters for different *Haemonchus contortus* isolates and the orthologous *Caenorhabditis elegans cyp-13A11* and *cyp-13A12*.

	ISE Reference ^a^	ISE ^b^	McM ^c^	WR ^d^	BSI ^e^	*C. elegans* *cyp-13A11* ^f^	*C. elegans* *cyp-13A12* ^g^
No AA ^h^	517	517	517	515	517	517	518
MW [kDa] ^h^	59.56	59.39	59.58	59.39	59.58	59.47	59.08
Theo. pI ^h^	8.76	8.97	8.97	9.09	8.62	6.36	6.08
Cyp heme motif ^i^	454–463 ^fj^: FGlGPRQCIG	454–463 ^j^: FGlGPRQCIG	454–463 ^j^: FGlGPRQCIG	452–461 ^j^: FGlGPRQCIG	454–463 ^j^: FGlGPRQCIG	454–463 ^j^: FGlGPRQCIG	455–464 ^j^: FGlGPRQCIG

^a^ Inbred-Susceptible Edinburgh (MHco3) reference. Sequence published by Doyle et al. [42] as HCON_00141052 (Accession no. LS997566, BioProject PRJEB506). ^b^ Inbred-Susceptible Edinburgh (MHco3). ^c^ McMaster. ^d^ White River. ^e^ Berlin-selected Isolate. ^f^ Wormbase ID: F14F7.2.1. ^g^ Wormbase ID: F14F7.3.1. ^h^ Number of amino acids (No AA), molecular weight (MW), and theoretical pI (Theo. pI) predicted by ExPASy–ProtParam (https://web.expasy.org/protparam/ accessed on 19 February 2021). ^i^ Cytochrome P450 cysteine heme-iron ligand (Cyp heme) motif predicted by ExPASy–ScanProsite (https://prosite.expasy.org/scanprosite/ accessed on 19 February 2021). ^j^ Motif position within the amino acid sequence.

**Table 3:** Docking characteristics of IVM, IVMa, SEL, EPR, DRM, and MOX in the human Cyp-3A4 (PDB: 6MA7) structure and Hco-Cyp-13A11 homology models. The docking energy was computed with AutoDock Vina (version 1.1.2) tools. The docking values in bold represent docking at the surface of the protein, as no docking into the active site could be simulated. The solvent-accessible surface area for the best docking position was estimated using PyMol. The number of hydrogen (H) bonds and hydrophobic interaction sites were determined with the web tool PLIP [61].

	Cyp-3A4 (PDB: 6MA7)	Hco-Cyp-13A11			
		ISE ^a^	McM ^b^	WR ^c^	BSI ^d^
**Ivermectin**					
Docking energy [kcal/mol]	−10.1	−10.5	−8.5	−6.5	−8.1
Solvent accessible surface Area [Å^2^]	993	1084	1058	962	1076
Number of H-bonds	3	2	4	8	2
Hydrophobic interactions	22	11	12	13	12
**Ivermectin aglycone**					
Docking energy [kcal/mol]	−8.6	−11.0	−11.3	−10.5	−11.2
Solvent accessible surface Area [Å^2^]	745	756	762	753	745
Number of H-bonds	1	3	2	1	3
Hydrophobic interactions	7	9	2	7	10
**Selamectin**					
Docking energy [kcal/mol]	−0.5	−8.2	−9.3	−9.1	−8.8
Solvent accessible surface Area [Å^2^]	949	953	918	918	927
Number of H-bonds	1	1	2	6	3
Hydrophobic interactions	15	11	13	11	11
**Doramectin**					
Docking energy [kcal/mol]	−4.1	−10.6	−8.6	**−11.1**	**−9.8**
Solvent accessible surface Area [Å^2^]	1139	1111	1134	**1148**	**1136**
Number of H-bonds	3	3	4	**3**	**4**
Hydrophobic interactions	16	13	9	**10**	**4**
**Eprinomectin**					
Docking energy [kcal/mol]	**−8.4**	−8.0	−7.7	**−8.5**	**−10.8**
Solvent accessible surface Area [Å^2^]	**1057**	1143	1068	**1104**	**1155**
Number of H-bonds	**4**	3	7	**3**	**4**
Hydrophobic interactions	**5**	15	15	**4**	**7**
**Moxidectin**					
Docking energy [kcal/mol]	−1.6	−11.0	−5.9	−8.9	−7.8
Solvent accessible surface Area [Å^2^]	875	870	881	870	876
Number of H-bonds	4	1	3	5	3
Hydrophobic interactions	13	10	7	8	6

^a^ Inbred-susceptible isolate. ^b^ McMaster isolate. ^c^ White River isolate. ^d^ Berlin-selected isolate.

## Data Availability

Plasmids are available upon request. The data from this study are available on request from the corresponding author.

## Supplementary Materials

The following are available online at https://www.mdpi.com/article/10.3390/ijms23169155/s1.
