# Supplementary material for: Transgenic Expression of Haemonchus contortus Cytochrome P450 Hco-cyp-13A11 Decreases Susceptibility to Particular but Not All Macrocyclic Lactones in the Model Organism Caenorhabditis elegans
Source: Int J Mol Sci. 2022 Aug 15;23(16):9155. doi: 10.3390/ijms23169155 (PMC9409383; doi:10.3390/ijms23169155)
Supplement: Supplementary file 1 [file ijms-23-09155-s001.zip › ijms-1768408-supplementary.pdf]

Transgenic expression of *Haemonchus contortus* cytochrome P450 *Hco-cyp-13A11* decreases susceptibility to particular but not all macrocyclic lactones in the model organism *Caenorhabditis elegans*

Natalie Jakobs, Esra Yilmaz and Jürgen Krücken *

Institute for Parasitology and Tropical Veterinary Medicine, Freie Universität Berlin, 14163 Berlin, Germany; nataliejakobs@web.de (N.J.); esrayz@msn.com (E.Y.)

*** Correspondence: juergen.kruecken@fu-berlin.de; Tel.: +49 (0) 30 838 62318**

Supplementary Materials: The following are available online at www.mdpi.com/xxx/s1, Figure S1: Vector design of expression plasmid., Figure S2: Comparison of thrashes per minute between the N2 and the transgenic *Hco-cyp-13A11 C. elegans*. Figure S3: Multiple sequence alignment of *Hco-cyp-13A11* amplified coding DNA sequences obtained by assembling exon regions from the genomic sequences., Figure S4: B-factor diagrams of Hco-Cyp-13A11 homology models represented by the B-factor putty program in PyMOL., Figure S5: Molecular docking analysis of Cyp-3A4., Figure S6: Molecular docking analysis of inbred-susceptible isolate Hco-Cyp-13A11., Figure S7: Molecular docking analysis of McMaster Hco-Cyp-13A11., Figure S8: Molecular docking analysis of White River Hco-Cyp-13A11.
Table S1: Primer sequences used for RT-PCR and assembly of plasmid construct., Table S2: Primer sequences used to amplify *Hco-cyp-13A11* exon encoding genomic regions., Table S3: Template alignment and sequence analysis of Hco-Cyp-13A11., Table S4: Parameters for evaluation of the best DOPE score model generated by Modeller., Table S5: Predicted positions of human Cyp-3A4 involved in substrate and heme cofactor binding and corresponding amino acids for Cyp-13A11 in different *Haemonchus contortus* isolates from amino acid sequence alignment and molecular docking of the heme cofactor., Table S6: Amino acids interacting with different macrocyclic lactones from molecular docking analysis.

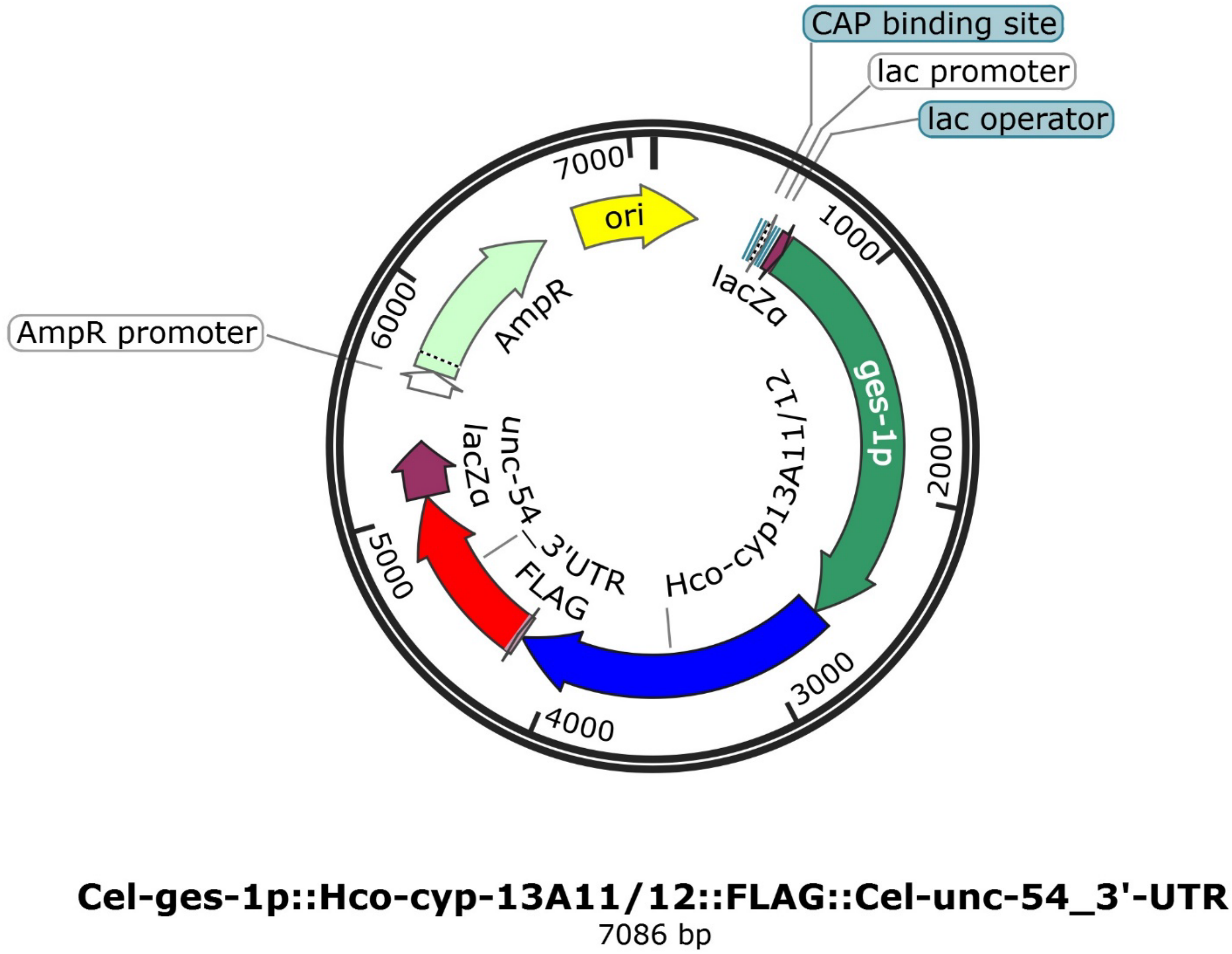

Figure S1: Vector design of expression plasmid. Vector map of plasmid driving extrachromosomal *Hco-cyp-13A11* expression generated with SnapGene (version 5.3). The pUC19 vector backbone contains elements for bacteria-specific replication and selection. *ges-1p*: *C. elegans ges-1* promoter; *Hco-cyp-13A11*: HCON_00141052, BioProject PRJEB506; *unc-54_3'UTR*: *C. elegans unc-54 3'-UTR* (untranslated region); FLAG: Flag-tag GAT TAT AAA GAT GAC GAT GAC AAA or DYKDDDDK (protein sequence).

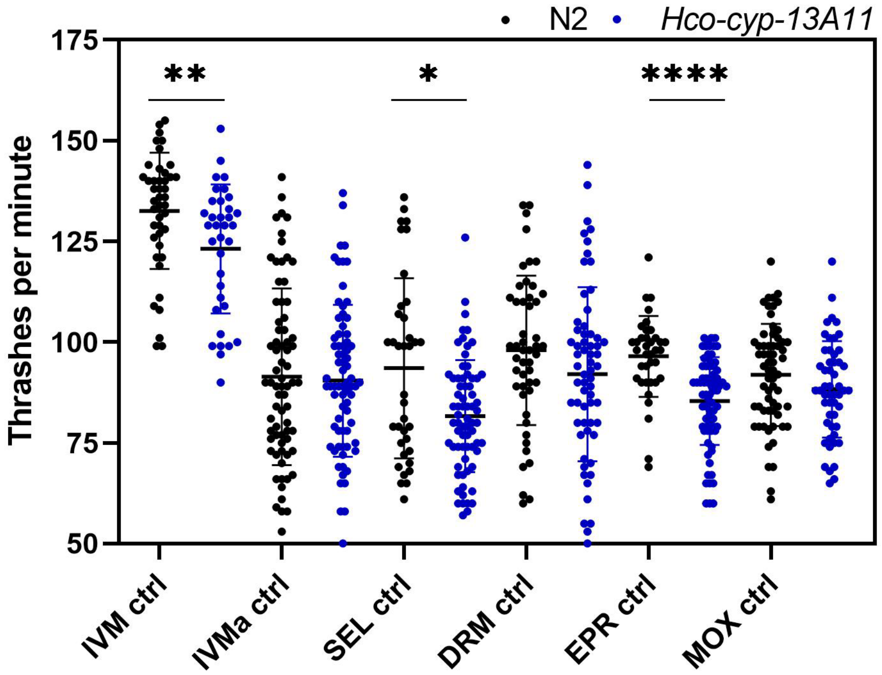

Figure S2: Comparison of thrashes per minute between the N2 and the transgenic *Hco-cyp-13A11 C. elegans*. The Mann Whitney *U* test was used to compare the thrashes per minute from all DMSO N2 controls (**black**) versus all DMSO controls of the transgenic *Hco-cyp-13A11 C. elegans* strain (**blue**) for each corresponding drug test (* $p = 0.0153$, ** $p = 0.0055$, **** $p<0.0001$). *Hco-cyp-13A11* genotype is *gutCyp-13A11Ex1 [Cel-ges-1p::Hco-cyp-13A11::FLAG::Cel-unc-54_3'-UTR; Cel-myo-2p::gfp::Cel-unc-54_3'UTR]*; control strain is N2 Bristol wild-type; ctrl: control; IVM: ivermectin; MOX: moxidectin; EPR: eprinomectin; IVMa: ivermectin aglycone; SEL: selamectin; DRM: doramectin.

```
WR               1 ATGCTTTTGCTTATCATCGCTGTGTCGTCACTTCTAGCTTTCATAAGCTATTACTACTGGCAACTCGATTACTGGAAGCGGCGAGGAATT
BSI              1 ATGCTTTTGCTTATCATCGCTGTGTCGTCACTTCTAGCTTTCATAAGCTATTACTACTGGCAACTCGATTACTGGAAGCGGCGAGGAATT
ISE              1 ATGCTTTTGCTTATCATCGCTGTGTCGTCACTTCTAGCTTTCATAAGCTATTACTACTGGCAACTCGATTACTGGAAGCGGCGAGGAATT
HCON_00141052    1 ATGCTTTTGCTTATCATCGCTGTGTCGTCACTTCTAGCTTTCATAAGCTATTACTACTGGCAACTCGATTACTGGAAGCGGCGAGGAATT
McM              1 ATGCTTTTGCTTATAATTGCTGTGTCGTCACTTCTAGCTTTCATAAGCTATTACTACTGGCAACTCGATTACTGGAAGCGGCGAGGAATT

WR              91 CCGGGTCCCCGAGGAATGATTTTTCTGGGAAACATGTATGAGCTGACAGATATATCTAAACCAGTCGGGTTGGTTTTGCGAGAGTGGACC
BSI             91 CCGGGTCCCCGAGGAATGATTTTTCTGGGAAACATGTATGAGCTGACAGATATATCTAAACCAGTCGGGTTGGTTTTGCGAGAGTGGACC
ISE             91 CCGGGTCCCCGAGGAATGATTTTTCTGGGAAACATGTATGAGCTGACAGATATATCTAAACCAGTCGGGTTGGTTTTGCGAGAGTGGACC
HCON_00141052   91 CCGGGTCCCCGAGGAATGATTTTTCTGGGAAACATGTATGAGCTGACAGATATATCTAAACCAGTCGGGTTGGTTTTGCGAGAGTGGACC
McM             91 CCGGGTCCCCGAGGAATGATTTTTCTGGGAAACATGTATGAGCTGACAGATATATCTAAACCAGTCGGGTTGGTTTTGCGAGAGTGGACC

WR             181 AAAACTTACGGCAAGGTTTACGGTATCCAAGAGGGATTGCGACGGACGATTGTCGTCAGCGATATTGAGATGATCCGCGAACTTTTCATA
BSI            181 AAAACTTACGGCAAGGTTTACGGTATCCAAGAGGGATTGCGACGGACGATTGTCGTCAGCGATATTGAGATGATCCGCGAACTTTTCATA
ISE            181 AAAACTTACGGCAAGGTTTACGGTATCCAAGAGGGATTGCGACGGACGATTGTCGTCAGCGATATTGAGATGATCCGCGAACTTTTCATA
HCON_00141052  181 AAAACTTACGGCAAGGTTTACGGTATCCAAGAGGGATTGCGACGGACGATTGTCGTCAGCGATATTGAGATGATCCGCGAACTTTTCATA
McM            181 AAAACTTACGGCAAGGTTTACGGTATCCAAGAGGGATTGCGACGGACGATTGTCGTCAGCGATATTGAGATGATCCGCGAACTTTTCATA

WR             271 AAAAAGTTTGATTATTTCTACGGTCGAAAGAGTGGCATCATTGGAGGTGATGTGGAGAAAGATTCTCGAGTTCATGTCTTTGAAGCACAA
BSI            271 AAAAAGTTTGATTATTTCTACGGTCGAAAGAGTGGCATCATTGGAGGTGATGTGGAGAAAGATTCTCGAGTTCATGTCTTTGAAGCACAA
ISE            271 AAAAAGTTTGATTATTTCTACGGTCGAAAGAGTGGCATCATTGGAGGTGATGTGGAGAAAGATTCTCGAGTTCATGTCTTTGAAGCACAA
HCON_00141052  271 AAAAAGTTTGATTATTTCTACGGTCGAAAGAGTGGCATCATTGGAGGTGATGTGGAGAAAGATTCTCGAGTTCATGTCTTTGAAGCACAA
McM            271 AAAAAGTTTGATTATTTCTACGGTCGAAAGAGTGGCATCATTGGAGGTGATGTGGAGAAAGATTCTCGAGTTCATGTCTTTGAAGCACAA

WR             361 GGTGTACGATGGAAACGCCTCAGAGCGATCTCTAGCCCTGCTTTTTCCTCGGGCTCTTTGAAGAAGATCCGCCCAACTGTGGAAAGCTCA
BSI            361 GGTGTACGATGGAAACGCCTCAGAGCGATCTCTAGCCCTGCTTTTTCCTCGGGCTCTTTGAAGAAGATCCGCCCAACTGTGGAAAGCTCA
ISE            361 GGTGTACGATGGAAACGCCTCAGAGCGATCTCTAGCCCTGCTTTTTCCTCGGGCTCTTTGAAGAAGATCCGCCCAACTGTGGAAAGCTCA
HCON_00141052  361 GGTGTACGATGGAAACGCCTCAGAGCGATCTCTAGCCCTGCTTTTTCCTCGGGCTCTTTGAAGAAGATCCGCCCAACTGTGGAAAGCTCA
McM            361 GGTGTACGATGGAAACGCCTCAGAGCGATCTCTAGCCCTGCTTTTTCCTCGGGCTCTTTGAAGAAGATCCGCCCAACTGTGGAAAGCTCA

WR             451 GTGCTTGCCCTGATGGAATTCTTTGAGAAAGAAGCCGACAACAAAGCTTTTGACATCTTCCCTTTTTATAAGGAGTTTACAATGGATGTG
BSI            451 GTGCTTGCCCTGATGGAATTCTTTGAGGAAGAAGCCGACAACAAAGCTTTTGACATTTTTCCTTATTATAAGGAGTTTACAATGGATGTG
ISE            451 GTGCTTGCCCTGATGGAATTCTTTGAGAAAGAAGCCGACAACAAAGCTTTTGACATTTTTCCTTTTTATAAGGAGTTTACAATGGATGTG
HCON_00141052  451 GTGCTTGCCCTGATGGAATTCTTTGAGACAGAAGCCGACAACAAAGCTTTTGACATTTTTCCTTTTTATAAGGAGTTTACAATGGATGTG
McM            451 GTGCTTGCCCTGATGGAATTCTTTGAGAAAGAAGCCGACAACAAAGCTTTTGACATTTTTCCTTTTTATAAGGAGTTTACAATGGATGTG

WR             541 ATTAGTCGCATCGCAATGGGTCAACAAGGTTCTCAGATGTTCAAGGACAAGAGGAAAGTGGCTACCATGGACTCCATATTTCGACGAAGT
BSI            541 ATTAGTCGCATCGCAATGGGTCAACAAGGTTCTCAAATGTTCAAGGACAAGAGGAAAGTGGCTACTATGGACTCCATATTCCGACGGAGT
ISE            541 ATTAGTCGCATCGCAATGGGTCAACAAGGTTCTCAGATGTTCAAGGACAAGAGGAAAGTGGCTACCATGGACTCCATATTTCGACGAAGT
HCON_00141052  541 ATTAGTCGCATCGCAATGGGCCAACAAGGTTCTCAGATGTTCAAGGACAAGAGGAAAGTGGCTACCATGGACTCCATATTTCGACGAAGT
McM            541 ATTAGTCGCATCGCAATGGGTCAACAAGGTTCTCAGATGTTCAAGGACAAGAGGAAAGTGGCTACCATGGACTCCATATTTCGACGAAGT

WR             631 TTACGGCAACCGATATTCTATTTGGCAAGCATGGCCCCATCGTTACGACGACCTCTGAGCCAGTTCTTCATGGCCATTGCTCCCCTGCGA
BSI            631 CTACGGCAACCGATATTCTTTTTGGCAAGCATGGCCCCATCGTTACGACGACCTCTGAGCCAGTTCTTCATGGCCATTGCTCCCCTGCGA
ISE            631 TTACGGCAACCGATATTCTATTTGGCAAGCATGGCCCCATCGTTACGACGACCTCTGAGCCAGTTCTTCATGGCCATTGCTCCCCTGCGA
HCON_00141052  631 TTACGGCAACCGATATTCTATTTGGCAAGCATGGCCCCATCGTTACGACGACCTCTGAGCCAGTTCTTCATGGCCATTGCTCCCCTGCGA
McM            631 TTACGGCAACCGATATTCTATTTGGCAAGCATGGCCCCATCGTTACGACGACCTCTGAGCCAGTTCTTCATGGCCATTGCTCCCCTGCGA

WR             721 AAGAGCAAAATTCCTGCACTTTTCAATGTTATTTACAAAACTATCGACGAGAGAATCGAGAAGAGGGCCCAAGACGCCGCGAAAGGTGTT
BSI            721 AAGAGCAAAATTCCTGCACTTTTCAATGTTATTTACAAAACTATCGACGAGAGAATCGAGAAGAGGGCCCAAGACGCCGCGAAAGGTGTT
ISE            721 AAGAGCAAAATTCCTGCACTTTTCAATGTTATTTACAAAACTATCGACGAAGGAATCGAGAAGAGGGCTCAAGACGCGGCGAAAGGTGTT
HCON_00141052  721 AAGAGCAAAATTCCTGCACTTTTCAATGTTATTTACAAAACTATCGACGAGAGAATCGAGAAGAGGGCCCAAGACGCCGCGAAAGGTGTT
McM            721 AAGAGCAAAATTCCTGCACTTTTCAATGTTATTTACAAAACTATCGACGAGAGAATCGAGAAGAGGGCCCAAGACGCCGCGAAAGGTGTT

WR             811 GTATCACAGGAGCACACTGACTTCATAGACCTCTTTTTGGACGCGAGAGCTGAACAGGACTTCGACAATAACGCCGAATTTTCAAAATTA
BSI            811 GTATCACAGGAGCACACTGACTTCATAGACCTCTTTTTGGACGCGAGAGCTGAACAGGACTTCGACAATAACGCCGAATTTTCAAAATTA
ISE            811 GTATCACAGGAGCACACTGACTTCATAGGCCTCTTTTTGGACGCGAGAGCTGAACAGGACTTCGACAATAACGCCGAGTTTTCAAAATTA
HCON_00141052  811 GTATCACAGGAGCACACTGACTTCATAGACCTCTTTTTGGACGCGAGAGCTGAACAGGACTTCGACAATAACGCCGAATTTTCAAAATTA
McM            811 GTATCACAGGAGCACACTGACTTCATAGACCTCTTTTTGGACGCGAGAGCTGAACAGGACTTCGACAATAACGCCGAATTTTCAAAATTA

WR             901 GGAGTTCAGGTTACAAAGCATTTGACGCGGGAAGAAATTGCTGCTCAGTGCTTCGTGTTTCTACTAGCGGGTTTCGACACTACTGCCACC
BSI            901 GGAGTTCAGGTTACAAAGCATTTGACGCGCGAAGAAATTGCTGCTCAGTGCTTTGTGTTTCTCCTGGCAGGCTTCGACACAACAGCTACC
ISE            901 GGAGTTCAGGTTACAAAGCATTTGACGCGCGAAGAAATTGCTGCTCAGTGCTTTGTGTTTCTCCTGGCAGGCTTCGACACAACAGCTACC
HCON_00141052  901 GGAGTTCAGGTTACAAAGCATTTGACGCGCGAAGAAATTGCTGCTCAGTGCTTTGTGTTTCTCCTGGCAGGCTTCGACACAACAGCTACC
McM            901 GGAGTTCAGGTTACAAAGCATTTGACGCGCGAAGAAATTGCTGCTCAGTGCTTTGTGTTTCTCCTGGCAGGCTTCGACACAACAGCTACC

WR             991 TCACTAGCATTCGTCACCTATCTGTTGGCGAAGAATCCTCGAGTTCAGCAAATTCTACAAGAAGAGATTGATCAGCACAG------TGAT
BSI            991 TCATTAGCATTCGTCACCTATCTGTTGGCGAAGAATCCTCGAGTTCAGCAGATTCTTCAAGAAGAGATTGATCAGCACTGCAACAGTGAC
ISE            991 TCATTAGCATTCGTCACCTATCTGTTGGCGAAGAATCCTCGAGTTCAGCAGATTCTTCAAGAAGAGATTGATCAGCACTGCAACAGTGAC
HCON_00141052  991 TCATTAGCATTCGTCACCTATCTGTTGGCGAAGAATCCTCGAGTTCAGCAGATTCTTCAAGAAGAGATTGATCAGCACTGCAACAGTGAC
McM            991 TCATTAGCATTCGTCACCTATCTGTTGGCGAAGAATCCTCGAGTTCAGCAGATTCTTCAAGAAGAGATTGATCAGCACTGCAACAGTGAC

WR            1075 AGTGAGACAATAAACTATGAGACTCTTTCAAGTATGAAGTATCTCGACTCCGTCATGAAGGAATCCTTGCGTATCTATCCTCTAGCAATC
BSI           1081 AGTGAGACAATCAACTATGAGACTCTCTCAAGTATGAAGTATCTCGACTCCGTCATGAAGGAATCCTTGCGTATCTACCCCCTAGCAGTC
ISE           1081 AGTGAGACAATCAACTATGAGACTCTCTCAAGTATGAAGTATCTCGACTCCGTCATGAAGGAATCCTTGCGTATCTACCCCCTAGCAGTC
HCON_00141052 1081 AGTGAGACAATCAACTATGAGACTCTCTCAAGTATGAAGTATCTCGACTCCGTCATGAAGGAATCCTTGCGTATCTACCCCCTAGCAGTC
McM           1081 AGTGAGACAATCAACTATGAGACTCTCTCAAGTATGAAGTATCTCGACTCCGTCATGAAGGAATCCTTGCGTATCTACCCCCTAGCAGTC

WR            1165 ATAGCTAACTCACGTCGCTGTATGAAATCGACCACGCTCGGACCGCTCAGGGTCGAAGAGGGTGAATTCGTAATGGTCGATACCATGTCT
BSI           1171 ATTGCTAACTCACGTCGCTGTATGAAATCGACCACGCTTGGACCGATCAGGGTCGAAGAGGGTGAATTCGTAATTGCCGATACCATGTCC
ISE           1171 ATTGCTAACTCACGTCGCTGTATGAAATCGACCACGCTTGGACCGATCAGGGTCGAAGAGGGTGAATTCGTAATTGCCGATACCATGTCC
HCON_00141052 1171 ATTGCTAACTCACGTCGCTGTATGAAATCGACCACGCTTGGACCGATCAGGGTCGAAGAGGGTGAATTCGTAATTGCCGATACCATGTCC
McM           1171 ATTGCTAACTCACGTCGCTGTATGAAATCGACCACGCTTGGACCGATCAGGGTCGAAGAGGGTGAATTCGTAATTGCCGATACCATGTCC

WR            1255 CTCCACTTTGATCGCGATCTTTGGGGAAACGATGCAGAGGAATTTAGACCTGAAAGATGGACTGAGTTGACGGCGAGGTCTTCAGCGGCG
BSI           1261 CTTCACTTTGATCGCGACATTTGGGGAGACGATGCAGAGGAATTTAGACCTGAAAGATGGACTGAGTTGACGGCGAGGTCTTCAGCGGCG
ISE           1261 CTTCACTTTGATCGCGACATTTGGGGAGACGATGCAGAGGAATTTAGACCTGAAAGATGGACTGAGTTGACGGCGAGGTCTTCAGCGGCG
HCON_00141052 1261 CTTCACTTTGATCGCGACATTTGGGGAGACGATGCAGAGGAATTTAGACCTGAAAGATGGACTGAGTTGACGGCGAGGTCTTCAGCGGCG
McM           1261 CTTCACTTTGATCGCGACATTTGGGGAGACGATGCAGAGGAATTTAGACCTGAAAGATGGACTGAGTTGACGGCGAGGTCTTCAGCGGCG

WR            1345 TTCTTGTCTTTCGGTCTTGGTCCAAGGCAGTGTATAGGAGTGCGACTAGCTCATATGGAAGAAAAGCTTGTTCTCGTTCATCTTCTCAGG
BSI           1351 TTCCTGTCTTTCGGTCTTGGTCCAAGGCAGTGTATAGGAATGAGACTAGCTCATATGGAAGAAAAGCTTGTTCTCGTTCATCTTCTCAGG
ISE           1351 TTCTTGTCTTTCGGTCTTGGTCCAAGGCAGTGTATAGGAGTGCGACTAGCTCATATGGAAGAAAAGCTTGTTCTCGTTCATCTTCTCAGG
HCON_00141052 1351 TTCTTGTCATTCGGTCTTGGTCCAAGGCAGTGTATAGGAATGCGACTAGCTCATATGGAGGAAAAGCTTGTTCTCGTTCATCTTCTCAGG
McM           1351 TTCTTGTCTTTCGGTCTTGGTCCAAGGCAGTGTATAGGAATGCGACTAGCTCATATGGAAGAAAAGCTTGTTCTCGTTCATCTTCTCAGG

WR            1435 CGGTATAACATCATTGCAACAAATGATACCGAGAAAGAACTGAAAATCAGAGGCTCGTTCACATTCGCTCCAGAAACTGTTACAGTCCAG
BSI           1441 CGGTATGACATCATTGCAACAAATGATACCGAGAAAGAACTGAAAATCAGAGGCTCGTTCACATTCGCTCCAGAAACTGTTACAGTCCAG
ISE           1441 CGGTATAACATCATTGCAACAAATGATACCGAGAAAGAACTGAAAATCAGAGGCTCGTTCACATTCGCTCCAGAAACTGTTACAGTCCAG
HCON_00141052 1441 CGGTATGACATCATTGCAACAAATGATACCGAGAAAGAACTGAAAATCAGAGGCTCGTTCACATTCGCTCCAGAAACTGTTACAGTCCAG
McM           1441 CGGTATAACATCATTGCAACAAATGATACCGAGAAAGAACTGAAAATCAGAGGCTCGTTCACATTCGCTCCAGAAACTGTTACAGTCCAG

WR            1525 CTACAACGAAGAGAACACTTATGA
BSI           1531 CTACAACGAAGAGAACACTTATGA
ISE           1531 CTACAACGAAGAGAACACTTATGA
HCON_00141052 1531 CTACAACGAAGAGAACACTTATGA
McM           1531 CTACAACGAAGAGAACACTTATGA
```

Figure S3: Multiple sequence alignment of *Hco-cyp-13A11* amplified coding DNA sequences obtained by assembling exon regions from the genomic sequences. Sequences were derived from different *Haemonchus contortus* isolates with HCON_00141052 (Accession no. LS997566, BioProject PRJEB506) as the reference sequence. Alignment was obtained using Clustal Omega (version 1.2.4) and shading created with BoxShade (version 3.21) with black letters presenting dissimilarities to the consensus sequence.

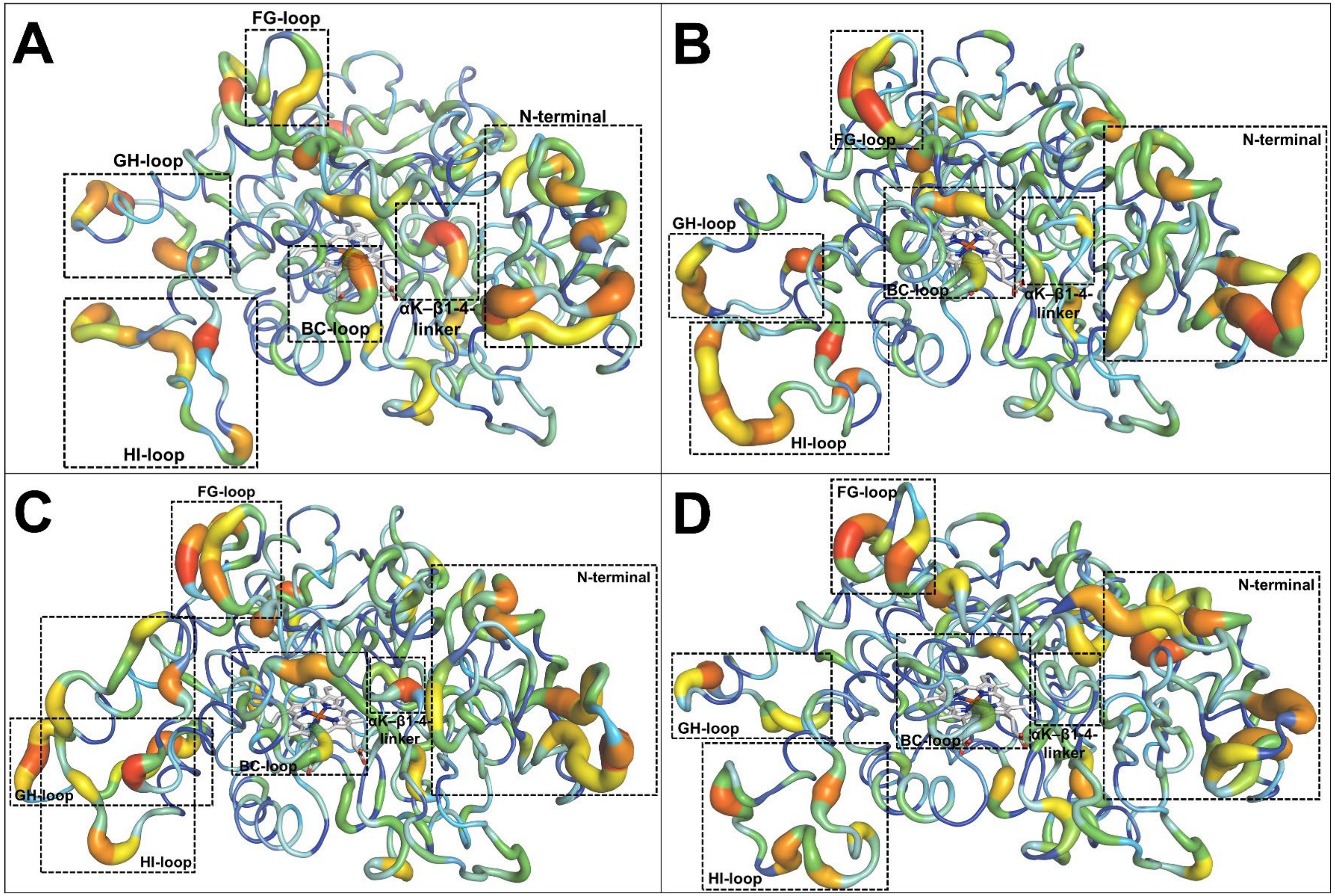

Figure S4: B-factor diagrams of Hco-Cyp-13A11 homology models represented by the B-factor putty program in PyMOL. **A** Inbred-susceptible Edinburgh. **B** McMaster. **C** Berlin-selected Isolate. **D** White River. The B-factor values are illustrated by color ranging from low (blue) to high (red) flexibility with highly flexible loops labeled.

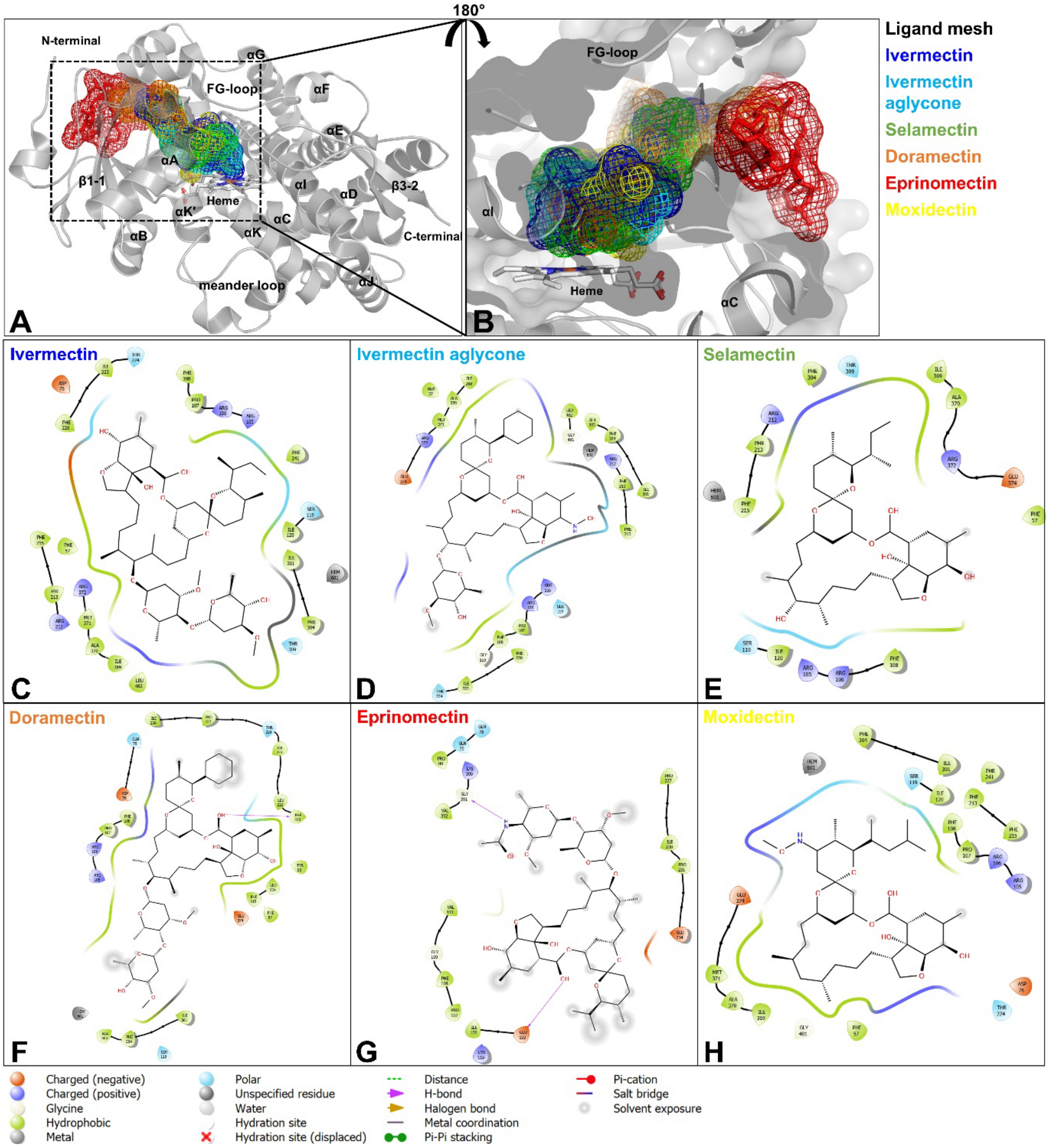

Figure S5: Molecular docking study of Cyp-3A4. **A, B** Overlayed structures of the human Cyp-3A4 (PDB: 6MA7) in complex with IVM (blue), IVMa (cyan), SEL (green), DRM (orange), EPR (red), and MOX (yellow). Ivermectin (**C**), IVMa (**D**), SEL (**E**), and MOX (**H**) docked to the active site cavity. DRM (**F**) and EPR (**G**) docked within a channel towards the active site. The 2D ML ligand-protein interaction diagrams show the closest residues within a 4 Å radius. 2D interaction plots were generated using Maestro Elements (version 4.6.117).

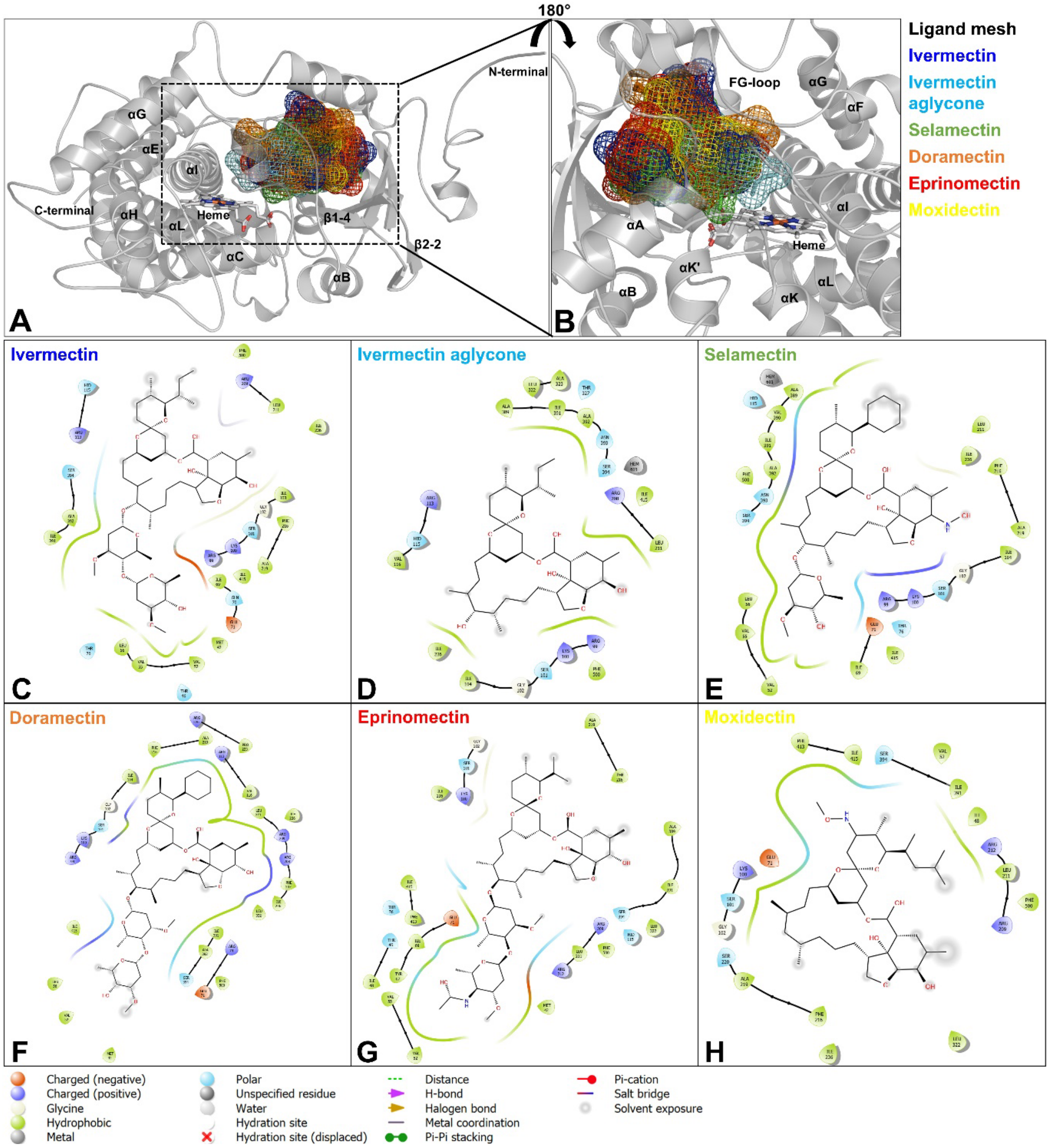

Figure S6: Molecular docking study of inbred-susceptible isolate Hco-Cyp-13A11. **A, B** Overlayed structures of the Hco-Cyp-13A11 Inbred-susceptible Edinburg isolate in complex with IVM (blue), IVMa (cyan), SEL (green), DRM (orange), EPR (red), and MOX (yellow) from different perspectives. IVM (**C**), IVMa (**D**), SEL (**E**), DRM (**F**), EPR (**G**) and MOX (**H**) docked to the active site cavity. The 2D ML ligand-protein interaction diagrams show the closest residues within a 4 Å radius. 2D interaction plots were generated using Maestro Elements (version 4.6.117).

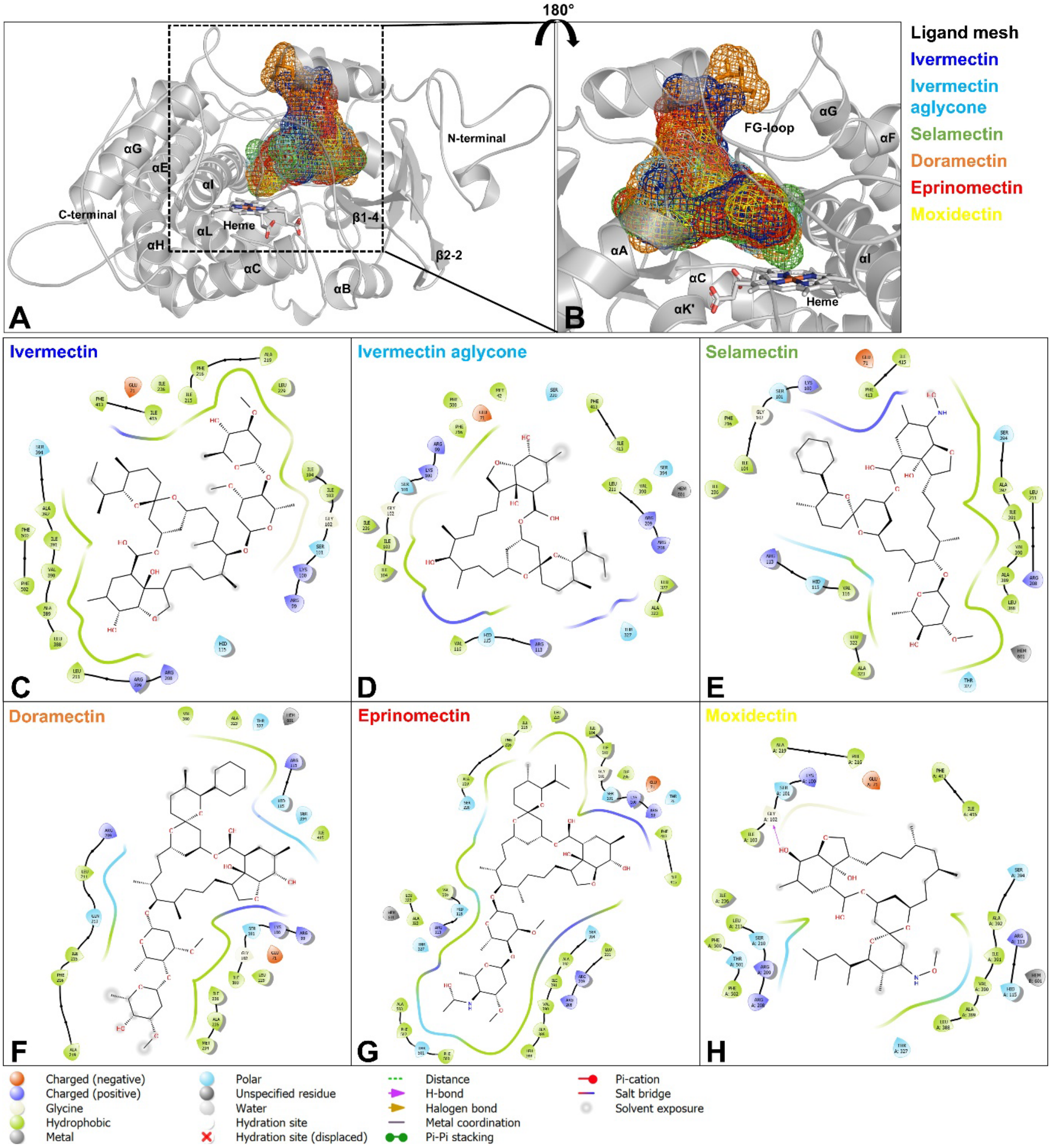

Figure S7: Molecular docking analysis of McMaster Hco-Cyp-13A11. **A, B** Overlayed structures of the Hco-Cyp-13A11 McMaster isolate in complex with IVM (blue), IVMa (cyan), SEL (green), DRM (orange), EPR (red), and MOX (yellow) from different perspectives. IVM (**C**), IVMa (**D**), SEL (**E**), DRM (**F**), EPR (**G**) and MOX (**H**) docked to the active site cavity. The 2D ML ligand-protein interaction diagrams show the closest residues within a 4 Å radius. 2D interaction plots were generated using Maestro Elements (version 4.6.117).

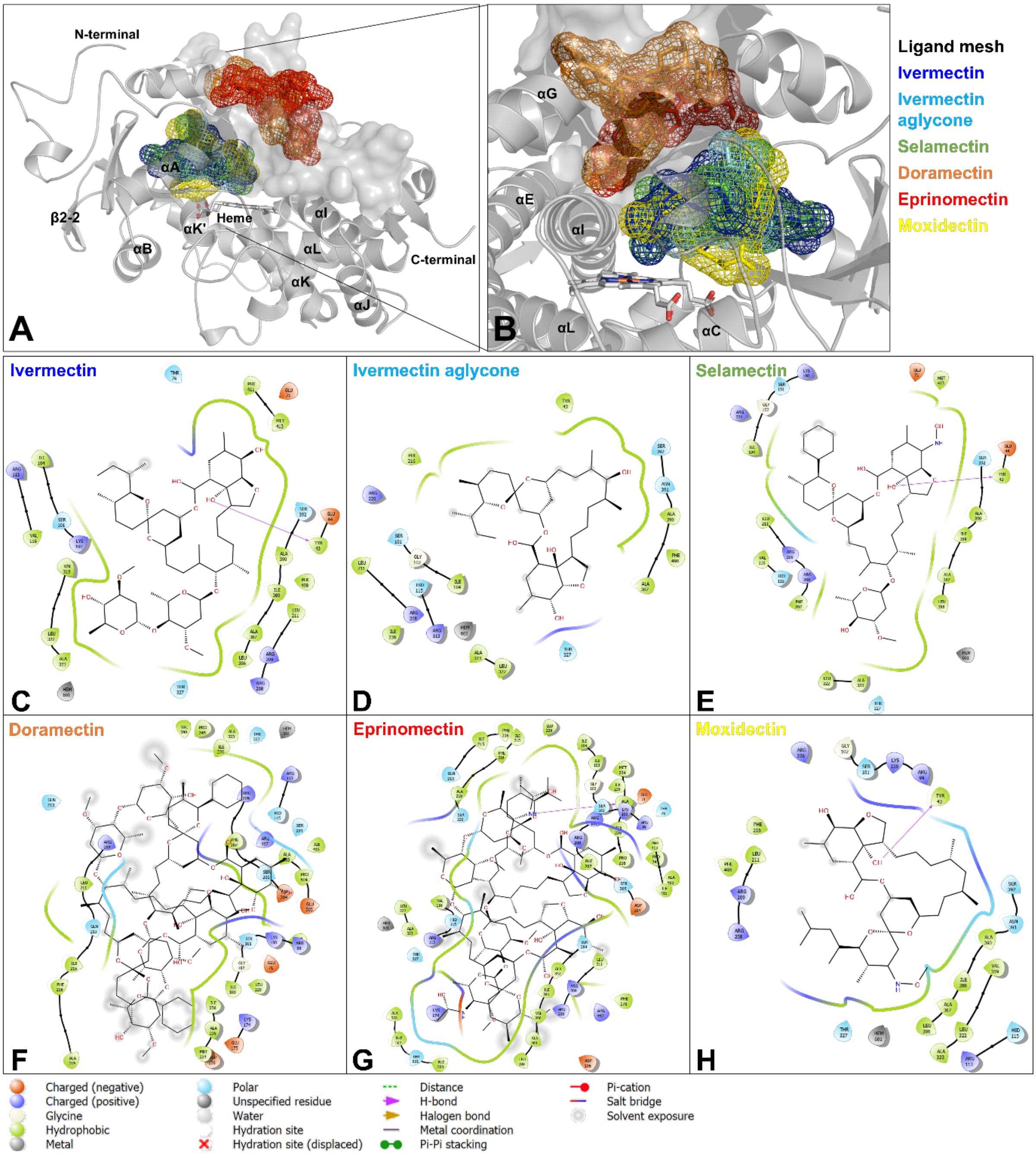

Figure S8: Molecular docking analysis of White River Hco-Cyp-13A11. **A, B** Overlayed structures of the Hco-Cyp-13A11 Berlin-selected isolate in complex with IVM (blue), IVMa (cyan), SEL (green), DRM (orange), EPR (red), and MOX (yellow) from different perspectives IVM (**C**), IVMa (**D**), SEL (**E**), and MOX (**H**) docked to the active site cavity. DRM (**F**) and EPR (**G**) showed the lowest binding energy docked at the outer surface of the FG-loop. The 2D ML ligand-protein interaction diagrams show the closest residues within a 4 Å radius. 2D interaction plots were generated using Maestro Elements (version 4.6.117).

Table S1: Primer sequences used for RT-PCR and assembly of plasmid construct. Assembly primers were designed using NEBuilder®Assembly Tool (version 2.3.1).

Purpose	Primer	Direction	Primer sequence (5'→3')	Size [bp]	Elongation time [s]	Annealing temperature [°C]
Assembly	pUC19_ges-1	fwd[a]	cgacggccagtgaattcgagctcggtacccAAACTCCGAACTATGATGAC	2000	30	55.6
	Hco-cyp-13A11_ges-1	rev[b]	gataagcaaaagcatCTGAATTCAAAGATAAGATATGTAATAG			
Assembly and RT-PCR	ges-1_Hco-cyp-13A11	fwd	tatctttgaattcagATGCTTTTGCTTATCATCG	1552	30	57.8
	unc54UTR_Hco-cyp-13A11	rev	catcgtcatctttataatcTAAGTGTTCTCTTCGTTGTAGC			
Assembly	Hco-cyp-13A11_unc54UTR	fwd	cgaagagaacacttagattataaagatgacgatgacaaatagGTCCAATTACTCTTCAACATC	848	30	55.1
	pUC19_unc54UTR	rev	atgcctgcaggtcgactctagaggatccccCCGGAAAACACAATAATG			

[a] fwd: forward.

[b] rev: reverse.

Table S2: Primer sequences used to amplify *Hco-cyp-13A11* (HCON_00141052, Accession no. LS997566, BioProject PRJEB506) exon encoding genomic regions to compare the sequences for different *Haemonchus contortus* isolates.

***Haemonchus contortus* isolate**	**(Overspanning) Exon region**	**Primer**	**Primer sequence (5'→3')**	**Amplicon length (bp)**	**Elongation time [s]**	**Annealing temperature [°C]**
White River	1, 2	fwd[a]<br>rev[b]	TGGTCACCTTTCGTCATCGG<br>GAATTCCTCGCCGCTTCCAG	1077	40	69.3
	2, 3, 4	fwd<br>rev	ACTTACAGCTATTACTACTGGCAA<br>TGTGAGTCAACGCACCTTCT	597	30	60.5
	5, 6, 7	fwd<br>rev	CTCTTTAGATCCGCCCAACTGT<br>TTTTAATCAACCAAACCTCAGAGG	501	30	62.4
	8, 9	fwd<br>rev	CGTTCTTGCCGTTCGATTGT<br>CGAGCTTGAACCGTTTTCTCG	429	30	64.4
	10, 11, 12	fwd<br>rev	GCCATCATTACAGGTTACAAAGCA<br>CCTTTCAGGTCTAAATTCCTCTGC	617	40	61.5
	13	fwd<br>rev	AGATGGACTGAGTTGACGGC<br>CACCTCGGTATCATTTGTTGCA	162	15	63.9
	14	fwd<br>rev	AGTCCTCGAAGTCTCGGTCA<br>CTCACGAGGAACAACCACCA	227	15	62.4
Berlin-Selected Isolate	1, 2	fwd<br>rev	TGGTCACCTTTCGTCATCGG<br>GAATTCCTCGCCGCTTCCAG	1077	40	68.2
	2, 3, 4	fwd<br>rev	AGTCAATAAAGTAGGTGGAGACGT<br>GCTTCGGTACCCCTGAAACC	694	30	63.0
	5, 6, 7	fwd<br>rev	CTCTTTAGATCCGCCCAACTGT<br>TTTTAATCAACCAAACCTCAGAGG	501	30	60.9
	8, 9	fwd<br>rev	CGTTCTTGCCGTTCGATTGT<br>CGAGCTTGAACCGTTTTCTCG	429	30	64.4
	10, 11, 12	fwd<br>rev	TGCTTCAGAAATGGCCATCA<br>GCACGATTCCTACCTCAGCA	603	40	60.5
	13	fwd<br>rev	AGATGGACTGAGTTGACGGC<br>CACCTCGGTATCATTTGTTGCA	162	15	62.4
	14	fwd<br>rev	AGTCCTCGAAGTCTCGGTCA<br>CTCACGAGGAACAACCACCA	227	15	61.9
Inbred-Susceptible Edinburgh	1, 2	fwd<br>rev	TGGTCACCTTTCGTCATCGG<br>GAATTCCTCGCCGCTTCCAG	1077	40	69.3
	2, 3, 4	fwd<br>rev	AGTCAATAAAGTAGGTGGAGACGT<br>GCTTCGGTACCCCTGAAACC	694	30	63.0
	5, 6, 7	fwd<br>rev	CTCTTTAGATCCGCCCAACTGT<br>TTTTAATCAACCAAACCTCAGAGG	501	30	60.9
	8, 9	fwd<br>rev	CGTTCTTGCCGTTCGATTGT<br>CGAGCTTGAACCGTTTTCTCG	429	30	64.4
	10, 11, 12	fwd<br>rev	TGCTTCAGAAATGGCCATCA<br>GCACGATTCCTACCTCAGCA	603	40	60.5
	13	fwd<br>rev	AGATGGACTGAGTTGACGGC<br>CACCTCGGTATCATTTGTTGCA	162	15	62.4
	14	fwd<br>rev	AGTCCTCGAAGTCTCGGTCA<br>CTCACGAGGAACAACCACCA	122	15	61.9
McMaster	1	fwd<br>rev	CTCTAGCGATACGACGGTG<br>AGTTCAAGGTACCTTATGAAAGCT	115	40	61.0
	2, 3, 4	fwd<br>rev	GGTTTGAAAGACTTACTTACAGCT ATT<br>TGTGAGTCAACGCACCTTCT	611	30	63.1
	5, 6, 7	fwd<br>rev	CTCTTTAGATCCGCCCAACTGT<br>TTTTAATCAACCAAACCTCAGAGG	501	30	60.9
	8, 9	fwd<br>rev	CGTTCTTGCCGTTCGATTGT<br>CGAGCTTGAACCGTTTTCTCG	429	30	63.3
	10, 11, 12	fwd<br>rev	TGCTTCAGAAATGGCCATCA<br>GCACGATTCCTACCTCAGCA	617	40	61.4
	13	fwd<br>rev	AGATGGACTGAGTTGACGGC<br>CACCTCGGTATCATTTGTTGCA	162	15	63.3
	14	fwd<br>rev	CCTCGAAGTCTCGGTCACAA<br>TGCTGTTCATAAGTGTTCTCTTCG	122	15	63.4

Table S3: Template alignment and sequence analysis of Hco-Cyp-13A11 for the isolates Inbred-Susceptible Edinburgh (ISE), McMaster (McM), White River (WR), and Berlin-Selected Isolate (BSI) used for single alignment modeling.

Protein	Organism	PDB ID	Resolution [Å]	Inhibitor[c]	Sequence identity (%)				E-value				Chain length
					ISE	McM	WR	BSI	ISE	McM	WR	BSI	
Cyp-3A4	*Homo sapiens*	6MA7[a]	2.09	Fluconazole	28.71%	28.92%	28.97%	28.92%	5e-58	5e-59	8e-60	1e-58	487
Cyp-3A4	*Homo sapiens*	4D6Z[a]	1.93	Tert-butyl {6-oxo-6-[(pyridin-3-ylmethyl)amino]hexyl} carbamate	29.32%	29.52%	29.58%	29.32%	4e-57	4e-58	6e-59	8e-58	487
Cyp-3A4	*Homo sapiens*	5A1R[b]	2.45	Progesterone	28.39%	28.60%	31.29%	31.22%	-	-	-	-	478

[a] The modeling templates were selected based on protein BLAST search (NCBI server).

[b] The modeling template was selected based on the SwissModel Template Search web tool (https://swissmodel.expasy.org/interactive). E-values are not given by the SwissModel Template Search web tool.

[c] Small molecule inhibitors bound the proteins when crystallized.

Table S4: Parameters for evaluating the best DOPE score model generated by Modeller (version 10.1) based on single alignment modeling and individual loop refinement.

	**Inbred-susceptible Edinburgh**[e]	**McMaster**	**White River**	**Berlin-selected Isolate**
**Modeller**				
Dope-Score	-58285	−59162	−58290	−57995
**Prosa-web**[a]				
Z-score	-7.66	−8.06	−7.64	−7.57
**Errat**[b]				
Overall quality factor	61.46	61.78	48.12	53.84
**QMEAN3**[c]				
QMEAN score	0.60 (Z-score: -5.24)	0.62 (Z-score: −4.72)	0.61 (Z-score: −4.79)	0.62 (Z-score: −4.49)
**ProCheck Ramachandran plot**[d]				
Residues in most favored regions	409 (87.0%)	422 (89.4%)	415 (88.3%)	419 (88.8%)
Residues in additional allowed regions	53 (11.3%)	39 (8.3%)	44 (9.4%)	45 (9.5%)
Residues in generously allowed regions	7 (1.5%)	10 (2.1%)	8 (1.7%)	5 (1.1%◎)
Residues in disallowed regions	1 (0.2%)	1 (0.2%)	3 (0.6%)	3 (0.6%)
Number of non-glycine and non-proline residues	470 (100.0%)	472 (100.0%)	470 (100.0%)	472 (100.0%)
Number of end-residues	2	2	2	2
Number of glycine residues	28	26	26	26
Number of proline residues	17	17	17	17

[a] Predicted by https://prosa.services.came.sbg.ac.at/prosa.php.

[b] Predicted by https://saves.mbi.ucla.edu/.

[c] Predicted by https://swissmodel.expasy.org/qmean/.

[d] Predicted by https://saves.mbi.ucla.edu/.

[e] The inbred-susceptible Edinburgh sequence was obtained by exon sequencing.

Table S5: Predicted positions of human Cyp-3A4 involved in substrate and heme cofactor binding and corresponding amino acids for Cyp-13A11 in different *Haemonchus contortus* isolates from amino acid sequence alignment and molecular docking of the heme cofactor.

Function	Cyp-3A4[a]	Hco-Cyp-13A11			
		ISE[b]	McM[c]	BSI[d]	WR[e]
Control of substrate specificity and regioselectivity (Sirim et al., 2010)	Ser119	Phe117	Phe117	Phe117	Phe117
Roof of the active site (Yano et al., 2004a)	Leu210	Ile206	Ile206	Ile206	Ile206
	Phe213	Arg209	Arg209	Arg209	Arg209
	Phe215	Leu211	Leu211	Leu211	Leu211
Allostery and stereoselectivity	Leu210	Ille206	Ille206	Ille206	Ille206
	Leu211	Phe207	Phe207	Phe207	Phe207
	Asp214	Ser210	Ser210	Ser210	Ser210
Position a water molecule for proton transfer (Yano et al., 2004a)	Glu308	Glu326	Glu326	Glu326	Glu326
	Thr309	Asp327	Asp327	Asp327	Asp327
Active site residues (Yano et al., 2004a)	Ser119	Phe117	Phe117	Phe117	Phe117
	Ile301	Phe319	Phe319	Phe319	Phe319
	Phe304	Phe322	Phe322	Phe322	Phe322
	Ala305	Leu323	Leu323	Leu323	Leu323
	Ile369	Leu388	Leu388	Leu388	Leu386
	Ala370	Ala389	Ala389	Ala389	Ala387
	Glu374	Ser394	Ser394	Ser394	Ser392
Coordination of Heme propionates (Williams, 2004) and polar interaction sites[f,g,h]	Arg105	*Arg99*	*Arg99*	*Arg99*	*Arg99*
	Ile118	*His115*	**Val114**	**Val114**	*His115*
	Trp126	Trp124	Trp124	Trp124	Trp124
	Arg130	**Arg128**	Arg128	Arg128	Arg128
	Arg375	*Asn393*	*Asn393*	*Asn393*	**Arg391**
	Ser437	Arg395	Arg395	Arg395	Arg393
	Arg440	**Phe454**	**Phe454**	**Phe454**	
		**Arg459**	Arg459	Arg459	Arg457
Heme-binding (Yano et al., 2004a)	Cys442	Cys461	Cys461	Cys461	Cys459

[a] Predicted position in 1TQN crystal structure (Yano et al., 2004b).

[b] Own Inbred-susceptible Edinburgh *H. contortus* sequence.

[c] McMaster isolate.

[d] Berlin-selected isolate.

[e] White River Isolate.

[f] Non-covalent polar interaction sites predicted by PyMOL in bold.

[g] Hydrogen Bonds computed by PLIP (Adasme et al., 2021) are underlined.

[h] Polar interaction sites predicted by PyMOL and PLIP in cursive.

Table S6: Residues and their corresponding regions within the protein forming hydrogen bonds with the substrates. Only interacting residues for ligand conformations with the lowest docking energy are listed for the human Cyp-3A4 and homology models of Hco-Cyp-13A11. The prediction was performed with the PLIP web tool (Adasme et al., 2021).

Drug	Region	Cyp-3A4 (PDB: 6MA7)	Hco-Cyp-13A11 Region	Inbred-susceptible Edinburgh	McMaster	White River	Berlin-selected isolate
**Ivermectin**		Asp76				Tyr43	
		Arg106				Thr76	
		Thr224					Lys100
					Gly102		
			BC-loop	Ile103			
						Arg113	Arg113
			FG-loop			Arg209	
					Leu388		
			αK-β1-4-linker			Ser392	
				Ser394	Ser394		
					Phe500		
**Ivermectin aglycone**		Arg105	BC-loop	Lys100			Lys100
					His115		
			αK-β1-4-linker			Ala390	
				Ser394	Ser394		Ser394
**Selamectin**	BC-loop	Ser119				Tyr43	
						Glu44	
							Lys100
			BC-loop	Gly102			Gly102
					His115	His115	
			FG-loop			Arg209	
			αK-β1-4-linker			Ser392	
					Ser394		
**Doramectin**	BC-loop	Ser119			Lys100		
		Phe220	BC-loop		Ile103		
		Thr224		Arg113			
			FG-loop	Arg209		Arg209	Arg209
							Gln213
			αK-β1-4-linker	Ser394	Ser394		
			β4-1			Arg495	Arg497
**Eprinomectin**		Gln79		Thr46			
		Glu122	BC-loop		Gly102		
	β2-2	Gly391	β3-1			Phe170	
					Arg208		Arg208
			FG-loop		Arg209		
							Ala235
			αI			Thr330	
			αK-β1-4-linker	Ala389			
				Ser394	Ser394		
			β4-1				Arg497
			β4-2		Phe502		
						Val505	
**Moxidectin**		Arg105				Tyr43	
		Arg106					Asp47
		Thr224					Lys100
			BC-loop			Ser101	
					Gly102		
					Arg113	Arg113	
			FG-loop				Arg209
						Arg226	
			αK-β1-4-linker	Ser394			
